# Phenotypic and Molecular Biological Analysis of Polymycovirus AfuPmV-1M From *Aspergillus fumigatus*: Reduced Fungal Virulence in a Mouse Infection Model

**DOI:** 10.3389/fmicb.2020.607795

**Published:** 2020-12-11

**Authors:** Azusa Takahashi-Nakaguchi, Erika Shishido, Misa Yahara, Syun-ichi Urayama, Akihiro Ninomiya, Yuto Chiba, Kanae Sakai, Daisuke Hagiwara, Hiroji Chibana, Hiromitsu Moriyama, Tohru Gonoi

**Affiliations:** ^1^Medical Mycology Research Center, Chiba University, Chiba, Japan; ^2^Faculty of Life and Environmental Sciences, University of Tsukuba, Tsukuba, Japan; ^3^Microbiology Research Center for Sustainability, University of Tsukuba, Tsukuba, Japan; ^4^Graduate School of Science, Technology and Innovation, Kobe University, Kobe, Japan; ^5^Department of Applied Biological Sciences, Tokyo University of Agriculture and Technology, Fuchu, Japan

**Keywords:** *Aspergillus fumigatus*, dsRNA, mycovirus, hypovirulence, mouse

## Abstract

The filamentous fungal pathogen *Aspergillus fumigatus* is one of the most common causal agents of invasive fungal infection in humans; the infection is associated with an alarmingly high mortality rate. In this study, we investigated whether a mycovirus, named AfuPmV-1M, can reduce the virulence of *A. fumigatus* in a mouse infection model. AfuPmV-1M has high sequence similarity to AfuPmV-1, one of the polymycovirus that is a capsidless four-segment double-stranded RNA (dsRNA) virus, previously isolated from the genome reference strain of *A. fumigatus*, Af293. However, we found the isolate had an additional fifth dsRNA segment, referred to as open reading frame 5 (ORF5), which has not been reported in AfuPmV-1. We then established isogenic lines of virus-infected and virus-free *A. fumigatus* strains. Mycovirus infection had apparent influences on fungal phenotypes, with the virus-infected strain producing a reduced mycelial mass and reduced conidial number in comparison with these features of the virus-free strain. Also, resting conidia of the infected strain showed reduced adherence to pulmonary epithelial cells and reduced tolerance to macrophage phagocytosis. In an immunosuppressed mouse infection model, the virus-infected strain showed reduced mortality in comparison with mortality due to the virus-free strain. RNA sequencing and high-performance liquid chromatography (HPLC) analysis showed that the virus suppressed the expression of genes for gliotoxin synthesis and its production at the mycelial stage. Conversely, the virus enhanced gene expression and biosynthesis of fumagillin. Viral RNA expression was enhanced during conidial maturation, conidial germination, and the mycelial stage. We presume that the RNA or translation products of the virus affected fungal phenotypes, including spore formation and toxin synthesis. To identify the mycovirus genes responsible for attenuation of fungal virulence, each viral ORF was ectopically expressed in the virus-free KU strain. We found that the expression of ORF2 and ORF5 reduced fungal virulence in the mouse model. In addition, ORF3 affected the stress tolerance of host *A. fumigatus* in culture. We hypothesize that the respective viral genes work cooperatively to suppress the pathogenicity of the fungal host.

## Introduction

*Aspergillus fumigatus* is a ubiquitous environmental fungus that is currently the most commonly encountered mold pathogen in severely immunocompromised patients. The emergence of drug-resistant fungi and the toxic side effects of antifungal drugs are two major problems associated with the treatment of fungal infections. Therefore, the development of new therapeutic strategies is urgently required. This may include the discovery of new chemotherapeutic drugs based on the identification of novel fungal targets and finding novel therapeutic methods for alleviating the pathogenic effects of fungi. One such hypothetical therapeutic strategy may include the use of mycoviruses, which can selectively infect pathogenic fungi.

Mycoviruses are viruses that selectively infect fungi and are widespread among all major taxa of fungi (Ghabrial and Suzuki, 2009; Ghabrial et al., 2015). The majority of characterized mycoviruses of plant-pathogenic fungi have double-stranded RNA (dsRNA) genomes. Mycoviruses with dsRNA genomes have been classified into eight families (the families *Totiviridae*, *Partitiviridae*, *Chrysoviridae*, *Amalgaviridae*, *Curvulaviridae*, *Reoviridae*, *Megavirnaviridae*, and *Polymycoviridae*; Several unsigned viruses including AfuPmV-1 have recently been established as a new family *Polymycoviridae* (Zhai et al., 2016; Jia et al., 2017; Kotta-Loizou and Coutts, 2017b; Niu et al., 2018; Zoll et al., 2018; Mahillon et al., 2019; Sato et al., 2020) (International Committee on Taxonomy of Viruses (ICTV)^1^. Although many mycoviruses do not appear to cause symptoms in their hosts, some affect mycelial growth, sporulation, and pigmentation, while in some botanical pathogenic fungi, mycoviruses have been shown to reduce their virulence in plant hosts (Pearson et al., 2009). However, our knowledge of mycoviruses in fungal pathogens infecting animals and humans remains limited (van de Sande et al., 2010; van de Sande and Vonk, 2019).

Screening of clinical and environmental *A. fumigatus* isolates for mycoviruses has revealed chrysovirus (AfuCV), partitivirus-1 (AfuPV-1), narnavirus-2 (AfuNV-1 and AfuNV-2), mitovirus-1 (AfuMV-1), and polymycovirus-1 (AfuPmV-1) in *A. fumigatus* strains (Bhatti et al., 2012; Kanhayuwa et al., 2015; Kotta-Loizou and Coutts, 2017a; Zoll et al., 2018). However, previous studies have reported that the introduction of AfuCV (Jamal et al., 2010) or AfuPV-1 virus (Bhatti et al., 2011) to host fungi provoked no significant alterations of pathogenicity in murine infection models. Two exceptions are: (1) infection with AfuPmV-1 virus showed slight potentiation in pathogenicity in an insect infection model (Kanhayuwa et al., 2015), and (2) our previous report of chrysovirus, a different viridae from the present work, showed reduced fungal virulence in a mouse model (Takahashi-Nakaguchi et al., 2020). Therefore, further mycoviruses that reduce *A. fumigatus* pathogenicity in a murine infection model remain to be detected (Özkan and Coutts, 2015).

The genome reference strain of *A. fumigatus*, Af293, contains a dsRNA mycovirus (Bhatti et al., 2012; Kanhayuwa et al., 2015). In this study, we report molecular biological and phenotypic analyses of a mycovirus that we detected in the Af293 strain. This virus exhibits high genome sequence similarity to the AfuPmV-1 virus (GenBank accession nos. HG975302–HG975305) but has a fifth dsRNA segment, which has not previously been described. Using a mouse infection model, we showed that the mycovirus-infected *A. fumigatus* strain possesses reduced virulence compared with that of the virus-free strain. We also discuss the roles played by each gene product in the attenuation of fungal pathogenicity, based on the findings of a gene expression experiment.

## Materials and Methods

### Isolates of *A. fumigatus* and Culture Methods

*A. fumigatus* strain Af293 was obtained from the American Type Culture Collection (ATCC), strain KU was obtained from the Fungal Genetics Stock Center (FGSC), and they were stored at Medical Mycology Research Center (MMRC), Chiba University. Fungi were grown on potato dextrose agar (PDA, Difco) at 37°C for 7 days until conidia were fully mature. To obtain mycelia for dsRNA extraction or virus purification, conidia were cultured in potato dextrose broth (PDB, Difco) at 37°C for 14 days with shaking at 180 rpm.

### Detection of dsRNA From Mycelia

Total nucleic acid was extracted from mycelia, and dsRNAs were purified by chromatography on CF-11 cellulose (Whatman) (Morris, 1979; Okada et al., 2013); any DNA or ssRNA segments were eliminated by treatment with DNase 1 (Takara Bio) and S1 nuclease (Takara Bio), respectively. The extracted dsRNAs were subjected to electrophoresis on 1% agarose gel in TAE buffer.

### cDNA Cloning and Phylogenetic Analysis

Five dsRNA segments and small fragments isolated from Af293 mycelia were fractionated by 5% (w/v) polyacrylamide gel electrophoresis and excised for further analysis. Double-stranded cDNA was synthesized from the dsRNAs using a cDNA Synthesis Kit (Roche). The cDNAs were then converted into an Illumina sequencing library, according to the manufacturer’s protocol (TruSeq RNA Sample Preparation Kit v2 -Set A, Illumina). Libraries were sequenced on an MiSeq (Illumina) as 250-bp paired-end reads. CLC Genomics Workbench (CLC bio.) was used to analyze the sequencing results. Sequences similar to those encoded by the dsRNA open reading frames (ORFs) were identified from the NCBI database using the BLASTn and BLASTp programs. Molecular phylogenetic analyses using the deduced amino acid sequences of the putative RdRp gene of AfuPmV-1M dsRNA1 were carried out using the CLUSTAL_X (Thompson et al., 1997) and MEGA 5 programs (Tamura et al., 2011). A bootstrap test was conducted with 1,000 resamplings for the neighbor-joining tree. Genome sequences of the five dsRNA segments isolated from Af293 were confirmed by Northern blot hybridization and RT-PCR. To obtain PCR clones that corresponded to the terminal region of each dsRNA, 5′RACE was used (5′Full RACE Core set; Takara Bio). Genome sequences for AfuPmV-1M were deposited in DDBJ/GenBank/EMBL, under the accession numbers LC517041–LC517045.

### Northern Blot Analysis

Samples of dsRNA (2-μg) were electrophoresed on 1% agarose–2.2 M formaldehyde gels and blotted onto Nylon Transfer Membrane (GE Healthcare). The hybridization probes used in this study were generated by PCR using the primers listed in Supplementary Table S3. Probes were labeled with AlkPhos Direct (GE Healthcare) and visualized using an LAS-1000 mini (Fujifilm co.).

### Relative Quantification of Viral RNA Expression Levels

For the synchronized induction of asexual development, conidia (10^5^ conidia/mL) were cultivated at 37°C in 20 mL PDB medium for 7 days, and conidia-free mycelia were harvested using Miracloth (Merck, Germany), washed with distilled water, and transferred onto PDA plates. The plates were then incubated at 37°C for specified times. The start of this plate incubation was referred to as 0 h, and mycelia were harvested at the time points of 0, 6, 12, 24, and 48 h.

Total RNA was isolated from *A. fumigatus* mycelium or conidia using an RNeasy Mini kit (Qiagen). Synthesis of cDNA from the total RNA was conducted with ReverTraAce using random primers (Toyobo, Japan). Subsequently, real-time PCR was conducted in 96-well plates with 20 μL reaction volumes containing THUNDERBIRD SYBR qPCR Mix (Toyobo). The samples were subjected to denaturation at 95°C for 30 s, followed by 40 cycles of amplification (95°C for 15 s, 60°C for 30 s) using a LightCycler 96 (Roche). Expression levels of viral RNA were normalized to the level of the constitutively expressed *A. fumigatus tef-1* gene, which served as an internal control (Gravelat et al., 2008). Primer sets are shown in Supplementary Table S4.

### Growth, Conidiation, and Germination Tests

To prepare fresh conidial suspensions, well-segregated conidia were inoculated onto a PDA slant and incubated at 37°C for 7 days. An appropriate volume of phosphate-buffered saline supplemented with 0.1% Tween 20 (PBST) was added and vortexed gently to obtain conidial suspensions. To test for colony growth, 10^5^ conidia of each strain were point-inoculated onto PDA plates, which were incubated at 37°C for 48 h. The numbers of conidia were counted using a hemocytometer. Counts were made in triplicate, and the mean values with standard deviations (SD) were reported. For analysis of germination, approximately 10^4^ conidia were incubated at 37°C in PDB. Germination was scored microscopically at 6 h.

### Scanning Electron Microscopy

For ultrastructural analysis of Af293 strains, conidia were cultured on PDA medium for 72 h, then fixed using the osmium vapor technique. Following fixation, samples were dried in a desiccator and deposited with platinum. All specimens were mounted on specimen stubs, sputter coated, and viewed under an S-3400N scanning electron microscope (HITACHI, Tokyo, Japan).

### Mycelial Dry-Weight Measurement

After culturing conidia of each strain in PDB at 37°C with rotation at 200 rpm for 24 h, the fungal communities were collected by filtration through a Miracloth (Merck Biosciences, United States). Following lyophilization, the dry weight of the cells was measured. The experiment was performed in triplicate.

### RNA-Seq

Total RNA was extracted from fungal cells using an RNeasy Mini Kit (Qiagen) and treated with DNase I (TaKaRa, Japan). Polyadenylated mRNA was then extracted from the total RNA and fragmented using a TruSeq RNA Sample Preparation Kit v2 – Set A (Illumina). A 200- to 300-nucleotide size selection was performed, and the RNA was then converted into an Illumina sequencing library according to the manufacturer’s protocol. Libraries were sequenced on a MiSeq sequencer (Illumina) as 50-bp single-end reads. The CLC Genomics Workbench ver. 12 (Filgen) was used to analyze the sequence results. Transcripts were categorized using FungiFun 2.2.8^2^. The sequence data have been deposited in the DDBJ/EMBL/GenBank database under the GEO accession number PRJDB9242.

### Detection of Gliotoxin and Fumagillin by HPLC

Conidia of AfuPmV-1M virus-infected and virus-free *A. fumigatus* (2.0 × 10^7^) were inoculated in 20 mL PDB and cultured for 6 days at 37°C with agitation at 120 rpm. Five independent culture experiments were performed for each strain. The culture supernatant was extracted using an equivalent volume of ethyl acetate. The mycelia were freeze-dried and extracted with 3 mL acetone. The extracts of the supernatant (5 mL) and the mycelia (1.5 mL) were dried *in vacuo* and dissolved in methanol. The solution was applied to an octadecylsilane column (Cosmosil 140C18-OPN, Nacalai Tesque, Inc., Kyoto, Japan) and eluted with methanol. The methanol fraction was dried *in vacuo* and dissolved in 100 μL DMSO. The DMSO solution was analyzed using a 1260 Infinity LC system (Agilent Technologies, Inc., Santa Clara, CA, United States) with a Poroshell 120 ECC18 column (ϕ3.0 mm × 100 mm, particle size 2.7 μm; Agilent). The LC analytical condition was a gradient elution of 5–100% acetonitrile containing 0.5% acetic acid for 18 min. Gliotoxin in the supernatant and fumagillin in mycelia were detected by absorbance at wavelengths of 254 and 330 nm, respectively.

### Virulence Assays in Mice

Six-week-old ICR male mice were supplied by Takasugi Experimental Animals Supply Company (Saitama, Japan). Mice were immunosuppressed with cyclophosphamide (Shionogi Pharmaceutics Co. Ltd., Osaka, Japan) at a dose of 25 mg kg^–1^ injected subcutaneously on days −2, 0, and 2 of conidial inoculation. The mice were housed in sterile cages with sterile bedding and provided with sterile feed and drinking water containing 300 mg/L tetracycline hydrochloride to prevent bacterial infection. The mice were intratracheally inoculated with 5 × 10^7^ conidia in 20 μL PBST on days 0 and 1. Mortality was monitored for 14 days, and statistical significance was assessed using the Kaplan–Meier log rank test.

The mice were euthanized, and their lungs were dissected at day 3 to determine fungal burdens by colony count and to facilitate histopathological examination (three mice per fungal strain). To count viable fungal cells, the lungs were weighed and homogenized using a Polytron homogenizer (PT1200E, KINEMATICA, Switzerland). The homogenate was spread over 9-cm PDA plates containing 0.05 mg/mL chloramphenicol. The plates were incubated at 37°C for 24 h and any colonies that formed were counted.

For the histopathological analysis, infected animals were euthanized on day 3 post-inoculation. The lungs were removed, fixed with formalin, and paraffin-embedded sections were stained with hematoxylin and eosin, as well as with Grocott’s methenamine silver. To compare the disease progress in the lungs of live mice, we used a third generation CT scanner, LaTheta LCT-200 (Hitachi-Aloka, Tokyo, Japan), on day 14. Prior to CT scanning, animals were anesthetized by inhalation of isoflurane (DS Pharma Animal Health Co., Ltd., Osaka, Japan) and maintained under isoflurane narcose during CT scanning.

### Adherence Assay

Cells of the type II human pneumocyte cell line A549 were obtained from ATCC and maintained in RPMI 1640 medium containing 10% fetal bovine serum (FBS, Gibco), 100 mg/L streptomycin, and 16 mg/L penicillin (both from Sigma). Cells were maintained at 37°C in a humidified 5% CO_2_ incubator. A549 cells were plated at 10^5^ cells/well in 6-well culture plates (BM Equipment Co., Ltd., Tokyo, Japan) and grown to confluence. The wells were overlaid with RPMI 1640 medium containing 100 conidia per well and incubated at 37°C under 5% CO_2_. After incubation for 2 h, wells were washed three times with PBS, overlaid with Sabouraud dextrose agar (SDA), and incubated at 37°C. After 24 h, conidia adhering to the wells were counted. In text, conidia adherence levels are expressed as the percentage ± SD of the number of adhering conidia divided by the original conidial number, based on three independent experiments.

### Cytotoxicity of *A. fumigatus* Against Cultured Cells

To measure the viability of mammalian cells in culture with the *A. fumigatus* strains, lactate dehydrogenase (LDH) levels were determined using the Cytotoxicity Detection KitPLUS (Roche). Samples were incubated with LDH substrate at 37°C for 5 min, then the absorbance was read on a microplate reader at 490 nm. Data were expressed as the percentage (±SD) of control cells without fungi, based on three independent experiments.

### Phagocytosis Assay

Murine macrophage-like cells (J774.1) were grown at a concentration of 10^5^ cells per well in RPMI 1640 medium supplemented with 10% (v/v) FBS and 100 mg/L streptomycin and 16 mg/L penicillin (both from Sigma) in 6-well plates overnight at 37°C under 5% (v/v) CO_2_. The cells were then co-incubated with 1 × 10^2^ *A. fumigatus* conidia for 2 h at 37°C under 5% CO_2_. To eliminate conidia not adhering to cells, the wells were washed with PBS and incubated for a further 5 h. The wells were overlaid with SDA and incubated at 37°C. Colony numbers were counted after 24 h incubation and were taken to represent the number of surviving conidia in the wells. In text, conidia survival rates are expressed as the percentage of the adhered conidial number divided by the overlaid conidial number.

### Measurement of Stress Responses

For assessing oxidative stress tolerance, 1 × 10^2^ conidia/mL were incubated with 32.6 mM H_2_O_2_ in PDB medium. After incubation for 1 h at 37°C, 100 μL samples of the suspensions were immediately plated onto SDA on 9-cm Petri dishes. After incubation for 24 h at 37°C, the number of visible colonies was counted. The growth ratio under oxidative stress was calculated by comparing the colony numbers on SDA for cells plated with and without H_2_O_2_. This procedure was based on a previously described method (Paris et al., 2003). To measure fungal growth under osmotic stress conditions, 10^6^ conidia of each strain were point-inoculated on yeast–glucose minimal medium (0.1% yeast extract–1% glucose, YGM), containing 0.8 M NaCl. After incubation for 72 h at 37°C, colony diameters were measured. The data are presented as the mean ± SD of three independent experiments.

## Results

### Nucleotide Sequences of dsRNAs

**FIGURE 1:**
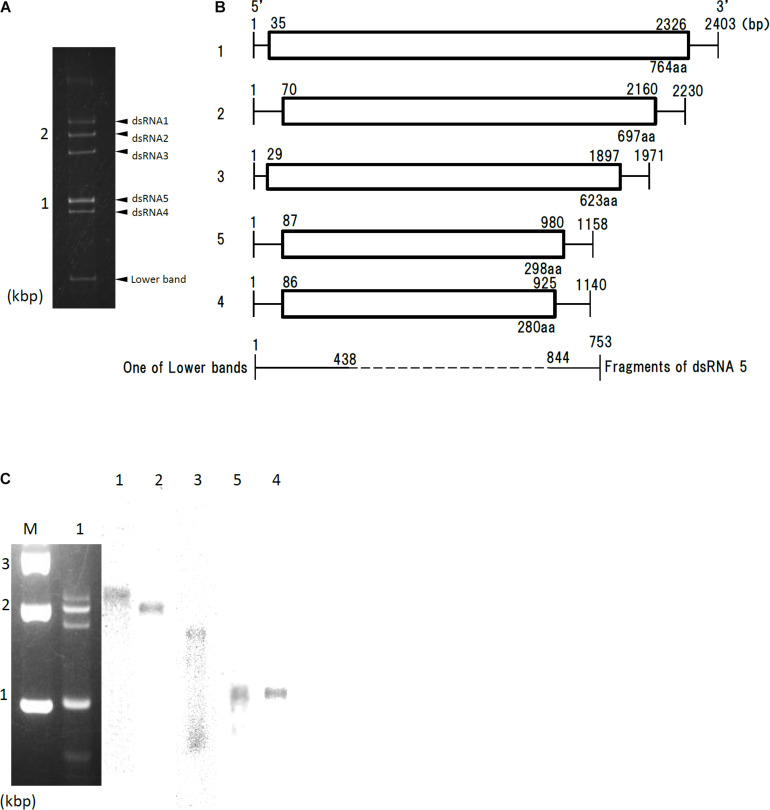
Genome organization of AfuPmV-1M. **(A)** dsRNA was extracted and electrophoresed on a 5% (w/v) polyacrylamide gel. **(B)** Schematic drawing of the genomic organization of AfuPmV-1M. “1”–“5” on the left indicates contigs. The horizontal lines and boxes indicate full dsRNA lengths and ORFs, respectively. The ORF sense orientations are from left to right for all contigs. Numbers on the lines and boxes indicate nucleotide numbers. Numbers under the boxes indicate amino acid (aa) lengths. **(C)** Left: Agarose gel electrophoresis of dsRNAs purified from the *A. fumigatus* strain infected with AfuPmV-1M (lane 1). Lane M, molecular weight marker. Right: Northern blot detection of dsRNA segments using digoxigenin-labeled DNA probes against dsRNA segments 1–5. dsRNA samples were electrophoresed on five different lanes of a single agarose gel and blotted. The five blotted lanes were separated and hybridized with the probes for each dsRNA. Lanes 1–5 of panel C were then reconstituted from the hybridized strips.

dsRNA extracted from Af293 cells was resolved into four bands by agarose gel electrophoresis, or five bands by polyacrylamide gel electrophoresis (Figure 1A, labeled segments 1 through 5). Sequences of the five dsRNAs were read and confirmed using a genome sequencer. Four of the five sequences had high sequence similarity to those of the previously reported AfuPmV-1 segments 1–4 (97.0, 96.8, 95.0, and 98.4% identity, respectively) (Özkan and Coutts, 2015; Kanhayuwa et al., 2015). We named the virus AfuPmV-1M as it was stocked at the Medical Mycology Research Center, Chiba, Japan. The new fifth dsRNA segment was then named AfuPmV-1M dsRNA 5. It should be noted that the AfuPmV-1M dsRNA 5 was slightly larger than dsRNA 4 of AfuPmV-1 and AfuPmV-1M (Figure 1B).

Northern hybridization analysis using DIG-labeled cDNA probes specific for each of the five dsRNA segments further confirmed that each segment had a unique sequence (Figure 1C). Sequence analysis of full-length cDNAs of segments 1–5 revealed they were 2,403, 2,330, 1,971, 1,158, (dsRNA 5), and 1,140 (dsRNA 4) bps (slightly different from those reported by Kanhayuwa et al., 2015 for AfuPmV-1) and that each segment contained a single open reading frame (ORF) (Figure 1B).

The 5′ and 3′ termini sequences in the five dsRNAs are shown in Supplementary Figure S1 and indicate high sequence conservation among these sequences. RNA sequencing of the Af293 strain was performed, and mapped to the genome of AfPmV1-M. The reads were mapped except for 1 to 2 bases at the very tip, which are difficult to map, indicating that the genome sequencing was correct. We also confirmed the results by viral RNA sequencing using fragmented and primer ligated dsRNA sequencing (FLDS; Urayama et al., 2016, 2018). This sequence conservation supports the idea that the five different segments belong to the same virus.

An examination of the deduced amino acid sequence of the AfuPmV-1M dsRNA 1 ORF revealed conserved motifs characteristic of RNA-dependent RNA polymerases (RdRp) of dsRNA viruses found in simple eukaryotes (Bruenn, 1993). A BLASTp search of the deduced amino acid sequence showed that it had high sequence similarity to RdRp encoded by Aspergillus fumigatus tetramycovirus-1 (AfuPmV-1, GenBank accession no. CDP74618.1; 98.4% identity), Botryosphaeria dothidea virus 1 (BdV-1, GenBank accession no. AKE49495.1; 54.4% identity), and Cladosporium cladosporioides virus 1 (CcV-1, GenBank accession no. YP_009052470.1; 46.0% identity). A phylogenetic tree based on RdRp sequences of AfuPmV-1M and 34 other selected RNA viruses was generated using the neighbor-joining (NJ) method (Saitou and Nei, 1987) and MEGA X (Tamura et al., 2011), indicating AfuPmV-1M belongs to the polymycoviridae dsRNA group (Supplementary Figure S2 and Supplementary Table S1).

A BLASTp search of the deduced amino acid sequence of AfuPmV-1M dsRNA 2 showed it had 99.6% identity with AfuPmV-1 dsRNA 2. It also showed sequence similarity to dsRNAs of BdV-1 (46.7% identity) and CcV-1 (32.3% identity). The functions of these proteins are unknown.

A MOTIF search^3^ of the deduced amino acid sequence of the AfuPmV-1M dsRNA 3 ORF revealed the presence of the conserved motif for methyltransferase from nucleotide positions 140 to 255, as seen in AfuPmV-1 from amino acids 110 to 250 (Kanhayuwa et al., 2015). A BLASTp search of the deduced amino acid sequence showed that it had sequence similarity to dsRNA 3 of AfuPmV-1 (97.9% identity) and the hypothetical proteins encoded by BdV-1 (42.8% identity) and CcV-1 (26.7% identity).

A BLASTp search of the deduced amino acid sequence of the AfuPmV-1M dsRNA 4 ORF showed that it had sequence similarity to dsRNA 4 of the PAS (proline/alanine/serine)-rich protein encoded by AfuPmV-1 (99.6% identity) and the hypothetical proteins encoded by BdV-1 (53.1% identity) and CcV-1 (39.8% identity).

Sequence analysis of AfuPmV-1M dsRNA 5 showed that it contained a single open reading frame (AfuPmV-1M dsRNA 5 ORF) from nucleotide positions 87 to 980. The dsRNA 5 ORF encodes a protein of 280 amino acid residues with a predicted molecular mass of 31 kDa (Figure 1B). A BLASTp search of the deduced amino acid sequence of dsRNA 5 revealed no significant sequence similarity with any virus proteins, while a BLASTn search revealed the nucleotide sequence from position 206 to 234 of dsRNA 5 had similarity to a conserved sequences of 29 nt in herpesviruses [CGTCGAGGACCCGTGGGCCCTGCCCGCGG (90% identity, GenBank accession number KP098534.1)].

During a series of experiments, bands of molecular weight lower than dsRNA 4 occasionally appeared following polyacrylamide gel electrophoresis (Figure 1A). Sequence analysis and Northern blotting (Figure 1C) revealed that one of these bands contained dsRNA 3 of AfuPmV-1M, as shown in Figures 1A,C, which lacked nucleotides from position 438 to 844 (resulting in a fragment that was 735 bp). Similar bands of less than 800 bp were occasionally observed during polyacrylamide gel analysis (data not shown). Sequence analysis revealed they contained 3′- and 5′-end parts of fragments 1 or 4 with missing central part similarly for the case of dsRNA 3, but they were not analyzed further in the present work.

### Expression Pattern of AfuPmV-1M Genes

**FIGURE 2:**
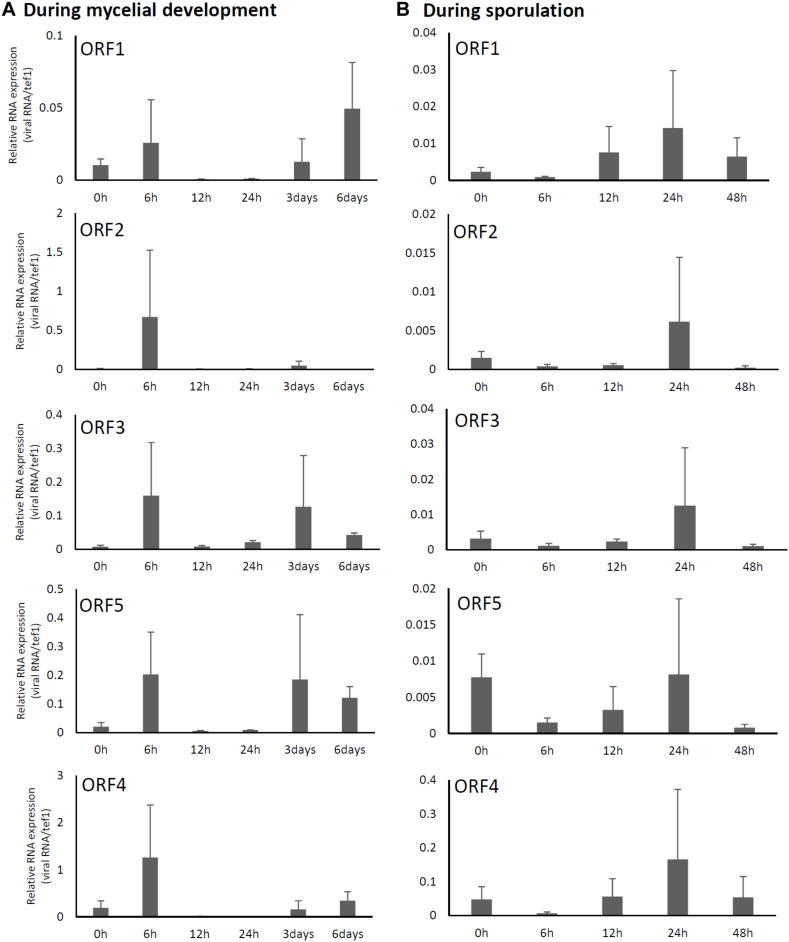
Changes in viral ORF transcript levels at different stages of growth in the AfuPmV-1M-infected strain. **(A)** RNA levels were analyzed by real-time PCR during the fungal life-cycle, with time points following exposure to liquid medium as follows: 0 h (conidia), 6 h (germination), and later stages of mycelial development (12 h, 24 h, and days 3 and 6). **(B)** RNA levels were analyzed using real-time PCR during sporulation, with time points as follows: 0 h (day 7, mycelia), 6 h (vesicle formation), 12 h (phialide formation), and 24 h and 48 h (conidial formation). Data values were normalized to that of the internal control, the *A. fumigatus* tef-1 gene, and presented as means ± SD of three independent experiments.

Relative expression levels of AfuPmV-1M genes in *A. fumigatus* were examined using real-time quantitative reverse transcription-PCR (qRT-PCR). Our results revealed that mycoviral RNA levels changed during the progression of fungal development. For all five AfuPmV-1M genes (dsRNAs 1–5), transcript levels were high at 6 h and 3–6 days after incubation in liquid medium; these time points corresponded to the germination stage of mycelial development and when mycelia were fully extended, respectively (Figure 2A). On the other hand, during sporulation (Figure 2B), transcript levels of the five ORFs were highest at a timepoint corresponding to the conidial maturation stage (24 h). ORF4 showed the highest expression level among the five ORFs (Figure 2).

### Effect of AfuPmV-1M Infection on *A. fumigatus* Colony Morphology and Mycelial Growth

To investigate whether the mycovirus was responsible for growth and virulence of *A. fumigatus*, we eliminated the mycovirus from the host using a single-spore isolation method. Elimination of the dsRNAs from the fungal hyphae was confirmed by dsRNA extraction, followed by agarose gel electrophoresis and ethidium bromide staining and RNA sequencing (Supplementary Figure S3).

**FIGURE 3:**
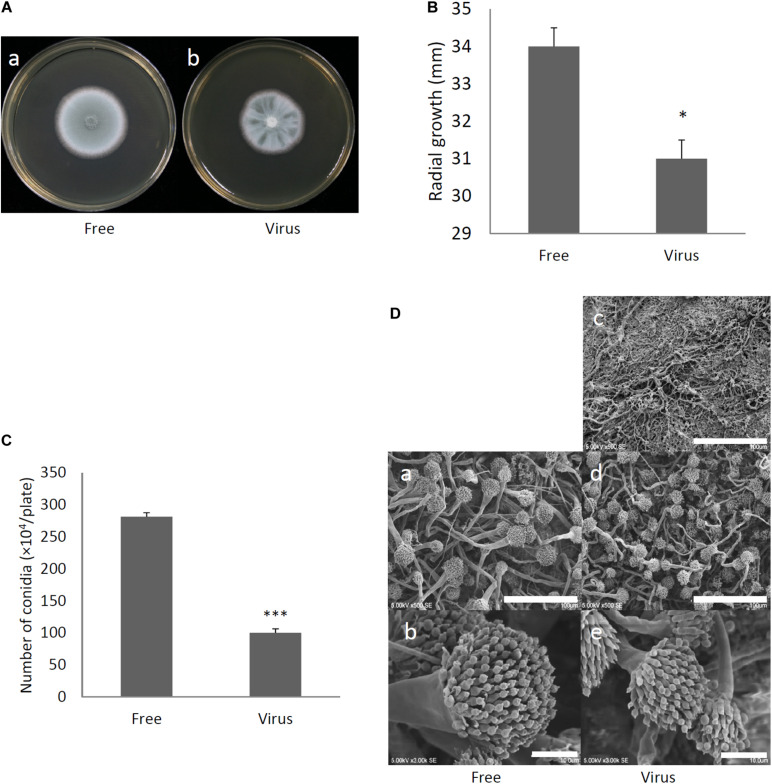
Comparisons of colony morphology and conidial formation of virus-free (Free) and virus-infected (Virus) strains. **(A)** Colony morphology of AfuPmV-1M virus-free **(a)** and virus-infected **(b)** *A. fumigatus* strains (cultured for 2 days). **(B)** Radial growth of colonies formed by virus-free and AfuPmV-1M virus-infected *A. fumigatus* strains. Statistically significance was determined using a two-tailed Student’s *t*-test, ^∗^*p* < 0.05. **(C)** Numbers of conidia formed by the strains at 24 h post-inoculation. Statistically significance, ^∗∗∗^*p* < 0.001. **(D)** Conidiophore formation in virus-free and virus-infected *A. fumigatus* strains. SEM images. **(a,b)** Virus-free. **(c–e)** Virus infected strain. **(a,d)** Green areas of the colonies. **(c)** White area of the colony. **(b,e)** Conidiophores. The scale bars indicate 100 μm for **(a,c,d)** and 10 μm for **(b,e)**.

**FIGURE 4:**
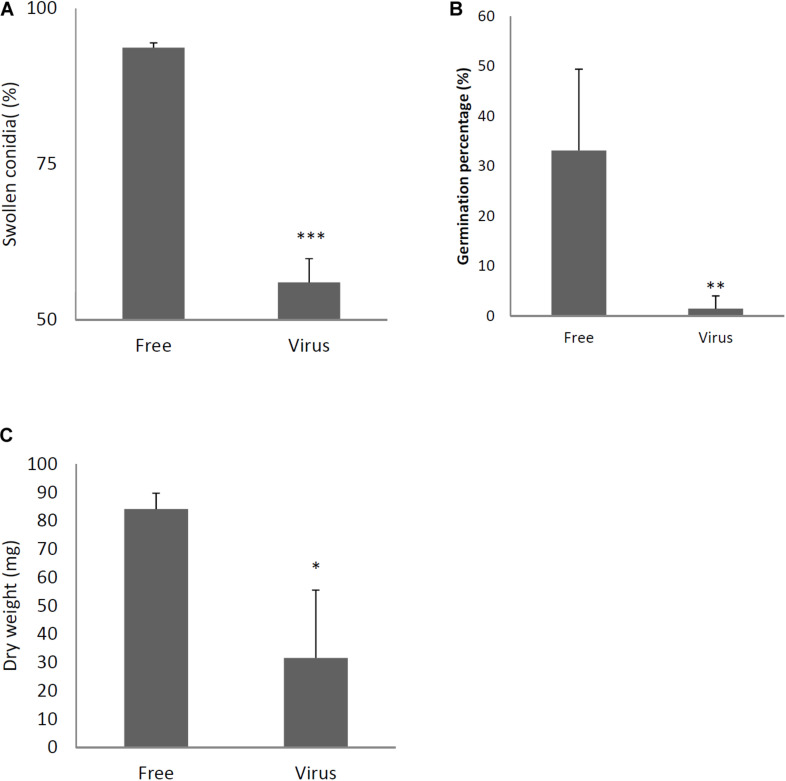
Comparisons of conidial swelling and germination rates and mycelial dry weight between the virus-free (Free) and virus-infected (Virus) strains. Percentage of swollen conidia at 6 h after plating **(A)** and percentage of germinating conidia at 8 h after plating conidia **(B)** are shown. **(C)** Mycelial growth. Growth was quantified by measuring dry weights of mycelia at 24 h after the incubation of conidia began. Data are presented as means ± SD of three independent experiments. ^∗^*p* < 0.05, ^∗∗^*p* < 0.01, ^∗∗∗^*p* < 0.001, by two-tailed Student’s *t*-test.

Two days after spotting conidia on potato dextrose agar (PDA) plates, the virus-free strain formed a colony of a homogeneous green color but the virus-infected strain formed a colony with stripes of green and white (Figure 3A). The difference in colony color had almost disappeared by day 3, when both colonies showed a homogenous green color. The virus-infected strain showed a reduced colony growth rate (Figure 3B), reduced numbers of conidiophores, and reduced conidia formation in comparison with the results of the virus-free strain (Figures 3C,D). During the *in vitro* experiments, conidia from the virus-infected strain showed delayed swelling and germination in comparison with these events in the virus-free strain (Figures 4A,B). Under liquid culture conditions in potato dextrose broth (PDB) medium, the mycelial mass of the virus-infected strain was also reduced (Figure 4C).

### Influence of AfuPmV-1M on *A. fumigatus* Gene Expression

To characterize how *A. fumigatus* responds to AfuPmV-1M infection, we performed RNA-seq analysis to compare gene expression between virus-infected and virus-free strains at two distinct time points: just before the start of swelling (4 h after the start of incubation, a stage referred to here as the 4-h swelling stage) and at the hyphal stage (day 6). At the 6-day hyphal stage, the pattern of host fungal gene expression differed more dramatically between the infected and the virus-free strains in comparison with gene expression at the 4-h swelling stage (4 h: *r* = 1.00, 6 days: *r* = 0.86, where “r” is the correlation coefficient) (Supplementary Figures S4A–C).

For gene expression assessed on day 6 after the start of incubation, 67 genes were down-regulated, and 198 genes were up-regulated, more than fivefold (Supplementary Table S2). There were some particular differences in virus-infected strains compared with the virus-free strains (Supplementary Figure S5 and Supplementary Table S2). In virus-infected strains, a series of genes in the fumagillin-related cluster increased (AFUA_8G00370 polyketide synthase, putative, AFUA_8G00380 DltD N-terminal domain protein, AFUA_8G00390 O-methyltransferase, putative, AFUA_8G00400 uncharacterized protein, AFUA_8G00430 uncharacterized protein, AFUA_8G00440 steroid monooxygenase, putative, AFUA_8G00480 phytanoyl-CoA dioxygenase family protein, AFUA_8G00500 acetate-CoA ligase, putative, AFUA_8G00510 cytochrome P450 oxidoreductase OrdA-like, putative, AFUA_8G00520 fumagillin beta-trans-bergamotene synthase, AFUA_8G00540 non-ribosomal peptide synthetase 14). Conversely, a series of genes in the gliotoxin-related cluster decreased (AFUA_6G09630 C6 finger domain protein GliZ, AFUA_6G09640 aminotransferase GliI, AFUA_6G09680 O-methyltransferase GliM, AFUA_6G09690 glutathione S-transferase GliG, AFUA_6G09700 gliotoxin biosynthesis protein GliK, AFUA_6G09710 MFS gliotoxin efflux transporter GliA, AFUA_6G09720 methyltransferase GliN, AFUA_6G09730 cytochrome P450 oxidoreductase GliF, AFUA_6G09740 thioredoxin reductase GliT, AFUA_6G09660 non-ribosomal peptide synthetase 10, gliotoxin synthesis protein). The quantities of fumagillin and gliotoxin in fungal cultures were measured by high-performance liquid chromatography (HPLC) and the results of this also supported the RNA-seq results. The amount of gliotoxin in supernatants was decreased, whereas the amount of fumagillin in mycelia was increased in virus-infected strains compared with the quantities in virus-free strains. In addition, gene ontology (GO) analysis showed that some RNA polymerase-related genes were down-regulated at both 4 h and 6 days (Supplementary Table S2).

### Hypovirulence of the dsRNA Virus-Infected *A. fumigatus* Strain in a Mouse Infection Model

**FIGURE 5:**
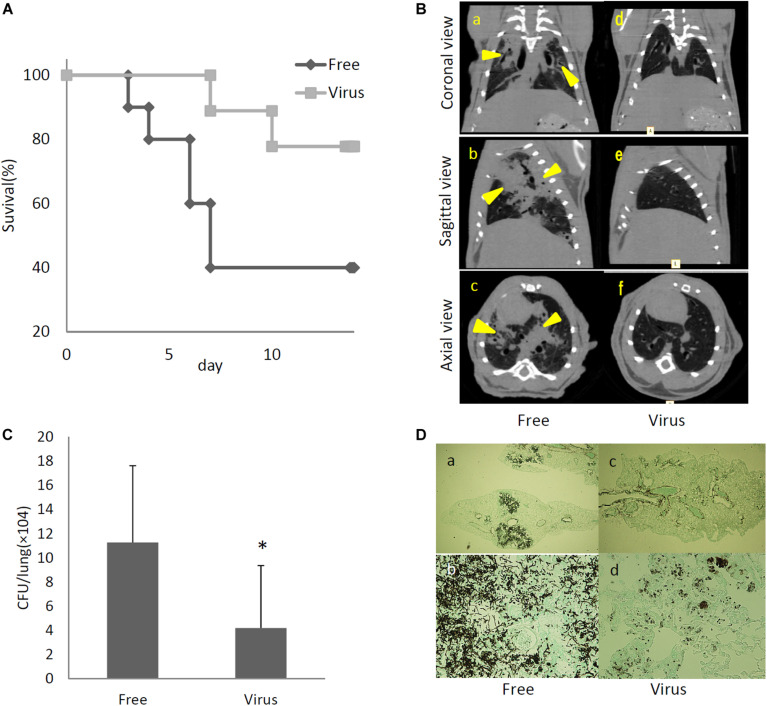
Comparisons of virulence in mice of virus-free (Free) and virus-infected (Virus) strains. **(A)** Survival rates of mice infected with the AfuPmV-1M virus-infected and virus-free strains. ^∗^*p* < 0.05 by the Kaplan–Meier log rank test. **(B)** CT scans of mouse chests at 14 days post-infection. **(a–c)** Virus-free fungal strain, **(d–f)** AfuPmV-1M virus-infected fungal strain. Shadows can be seen in the lungs of virus-free *A. fumigatus*-infected mice (arrowheads in **a–c**). **(C)** Fungal growth from the lungs of infected mice. Fungal burdens were estimated as CFUs per gram of lung. Data are presented as means ± SD of three independent experiments. ^∗^*p* < 0.05 by two-tailed Student’s *t*-test. **(D)** Lung histology of mice at 3 days post-infection (Gomori’s methenamine silver-staining-Grocott’s variation). **(a,b)** virus-free strains; **(c,d)** virus-infected strains.

To determine the effects of dsRNA virus infection on the virulence of *A. fumigatus*, virus-infected and virus-free strains were intratracheally administrated to ICR mice immunosuppressed with hydrocortisone acetate. As shown in Figure 5A, the survival rate was significantly higher in mice infected with the virus-infected *A. fumigatus* strain compared with the survival of those mice infected with the virus-free strain (*p* < 0.05, Kaplan–Meier log rank test). Computed tomography (CT) imaging was performed in mice infected with the virus-infected and virus-free strains on day 14 following infection. Mice infected with the virus-free *A. fumigatus* strain showed a dense consolidation of the lung, consistent with pneumonia, while mice infected with the virus-containing *A. fumigatus* showed no radiographic or pathologic evidence of pneumonia (Figure 5B). The pulmonary fungal burden at 72 h after fungal infection was significantly lower in mice infected with the virus-infected fungal strain than in mice infected with the virus-free strain (Figure 5C). The results of Grocott’s methenamine silver (GMS) staining of the lungs at 72 h after infection revealed that hyphae were observed in mice lungs infected with the virus-free *A. fumigatus* strain, but no hyphal growth was observed from conidia of the virus-infected strain (Figure 5D).

**FIGURE 6:**
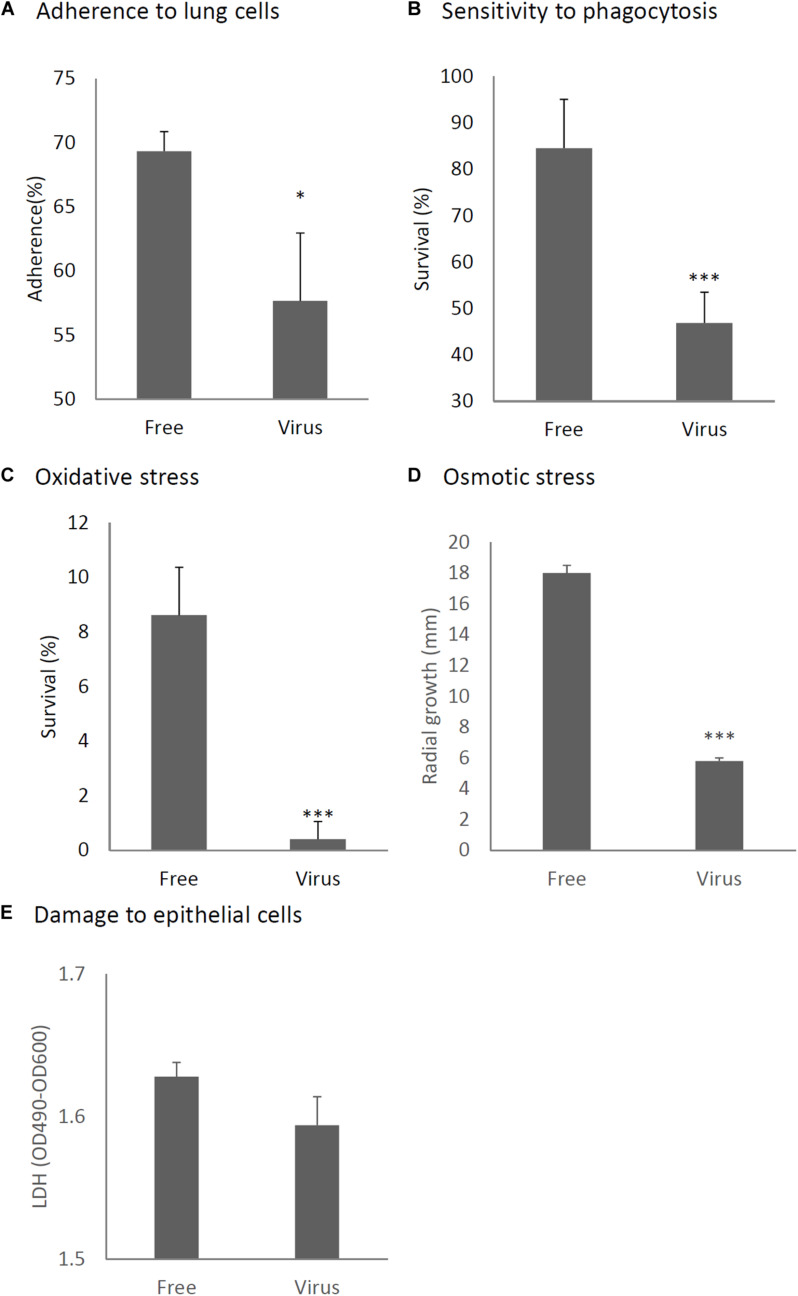
Comparison of phenotypes and tolerance to stresses between virus-free (Free) and virus-infected (Virus) strains. **(A)** Adherence of conidia to A549 human pneumocyte cells. **(B)** Sensitivity of the *A. fumigatus* strains to phagocytosis by J774A.1, a murine macrophage cell line. **(C)** Germination rates of the virus-free and virus-infected strains under oxidative stress (germination following 24-h exposure to 32.6 nM H_2_O_2_). **(D)** Growth of the virus-free and virus-infected *A. fumigatus* strains under osmotic stress conditions (conidial growth in the presence of 0.8 M NaCl). **(E)** Mammalian epithelial cell (A549) lysis after co-incubation with conidia. From **(A)** to **(E)**, all experiments were independently repeated three times. Mean ± SD. ^∗^*p* < 0.05, ^∗∗∗^*p* < 0.001, by two-tailed Student’s *t*-test.

We considered it possible that the reduction of virulence in the mycovirus-infected strain may be due to decreased adherence of the conidia to host epithelial cells or, alternatively, reduced survival rate of fungi after phagocytosis by macrophages in the lungs of mice. We therefore, studied the impact of the mycovirus on the conidial adherence of *A. fumigatus* to lung epithelial cells and on the survival rate of conidia of virus-infected or virus-free strains after *in vitro* culturing with macrophages. As shown in Figure 6A, conidia of the virus-infected strain showed reduced adherence to cells of the type II human pneumocyte cell line A549 in comparison with the adherence of the virus-free strain. Also, conidia from the virus-infected strain showed a reduced survival rate after co-culturing with mouse macrophage J774 cells compared with the survival rate of the virus-free strain (Figure 6B).

Previous reports have claimed that reactive oxygen species are manufactured in macrophages for the purpose of killing invading microorganisms (Weiss and Schaible, 2015). Our experiment showed the conidia of the virus-infected strain were more sensitive to oxidative stress caused by H_2_O_2_ in comparison with the virus-free strains (Figure 6C). Tolerance to osmotic stress was also decreased in the virus-infected strain (Figure 6D). An LDH (lactate dehydrogenase) assay was used to quantitatively assess epithelial cell lysis upon infection. No significant difference in epithelial damage was observed between the virus-infected and virus-free strains (Figure 6E).

### Phenotypic Analysis of Fungal Strains Expressing Individual ORF

**FIGURE 7:**
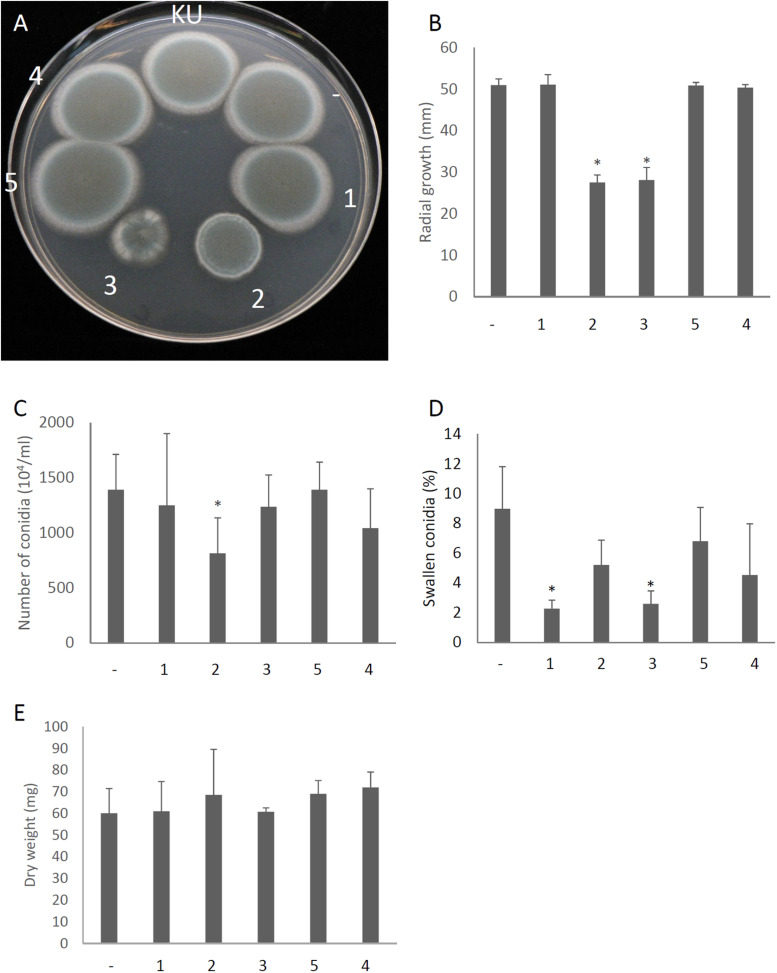
Morphological comparisons of AfuPmV-1M ORF expressing and non-expressing KU strains. **(A)** Colonies formed by native KU strain (KU), empty-vector expressing (−), and each AfuPmV-1M ORF-expressing KU strain (1–5). **(B)** Radial growth of colonies by ORF expressing (1–5) and non-expressing (−) KU strains. **(C)** Number of conidia formed by the strains at 3 days post- inoculation. **(D)** Percentages of swelling conidia at 6 h after the start of incubation. **(E)** Dry weight of mycelia. Mycelial growth was quantified by measuring their dry weight at 24 h after starting the incubation of conidia. Data are presented as means ± SD of three independent experiments. ^∗^*p* < 0.05, by one-way ANOVA, Dunnett’s test.

We attempted to identify which gene products of AfuPmV-1M are involved in hypovirulence by establishing *A. fumigatus* strains which express the individual viral ORFs. Each of the AfuPmV-1M ORFs was expressed in an originally virus-free strain (KU) using a pCB1004 plasmid vector. Viral gene expression in each transformants during sporulation were confirmed by qRT-PCR (Supplementary Figure S7). Compared with the KU strain or a strain transformed with the empty vector, ORF2- and ORF3-expressing strains showed significant changes in colony morphology, with radial growth being largely suppressed (Figures 7A,B). Expression of ORF2 also reduced the number of conidia formed after 3 days in culture (Figure 7C). Microscopic examination revealed that the numbers of swollen conidia were reduced in transformants expressing ORF1 and ORF3 (Figure 7D), while mycelial growth in liquid culture was not significantly changed by the expression of any of the ORFs (Figure 7E).

**FIGURE 8:**
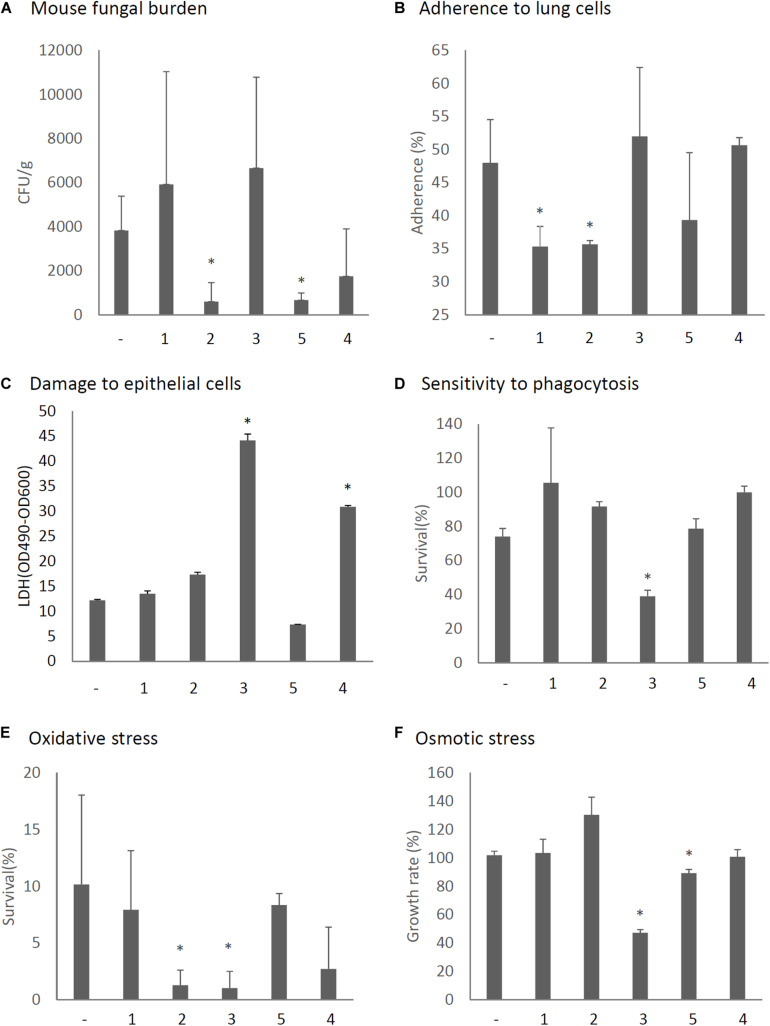
Phenotypic comparisons of each AfuPmV-1M ORF (1–5)-expressing and non-expressing (−) KU strain. **(A)** Fungal burden in mouse lungs. Fungal cell numbers in mouse lungs were estimated as CFU of lung tissues 3 days after infection with each fungal strain. **(B)** Adherence of conidia from each strain to A549 human pneumocyte cells. **(C)** Cellular cytotoxicity of each ORF expressing (1–5) and non-expressing (−) strain assayed by LDH activity. A549 cells were incubated with each strain for 24 h for the LDH assay. **(D)** Sensitivity of each strain to phagocytosis by J774A.1, a murine macrophage cell line. **(E)** Effect of hydrogen peroxide on the growth of each strain. **(F)** Growth of each strain under osmotic stress conditions. ^∗^*p* < 0.05, by one-way ANOVA, Dunnett’s test.

The virulence of *A. fumigatus* KU strain expressing each ORF or only the empty vector was then assessed in immunosuppressed ICR mice. As shown in Figure 8A, we observed that the expression of ORF2 and ORF5 decreased the fungal burden in the lungs (CFU/g), as observed 3 days after infection and compared with the empty vector control. *In vitro* assays revealed that conidia of ORF1- and ORF2-expressing strains showed reduced adherence to A549 cells in comparison with the adherence of the KU strain transformed with the empty vector (Figure 8B). Damage to epithelial cells was increased in the ORF4- and ORF5-expressing strains (Figure 8C). The survival rate of the fungi in co-culture with J774A.1 mouse macrophages decreased in the ORF3-expressing strain (Figure 8D). Tolerance to oxidative stress was decrease in the ORF2- and ORF3-expressing strains (Figure 8E), while tolerance to osmotic stress was decreased in the ORF3- and ORF5-expressing strains (Figure 8F).

## Discussion

It is possible that the study of mycoviruses could provide clinically useful information because the virus might cause hypovirulence in its fungal hosts. Although several mycoviruses are known to be associated with latent infections in human pathogenic fungi, few reports have described their effects on fungal pathogenicity against hosts. The present study revealed the AfuPmV-1M dsRNA mycovirus, carried by *A. fumigatus* strain Af293, causes hypovirulence in its host human pathogenic fungi. To the best of our knowledge, this is only the second report after one about AfuCV41362 (Takahashi-Nakaguchi et al., 2020) of a mycovirus causing hypovirulence of invasive fungal infection in mammals. It should be noted that AfuCV41362 is classified in the *Chrysoviridae*, while AfuPmV-1M, described here, is classified in the *Polymycoviridae*.

In previous studies, four separate viral dsRNA bands from the Af293 strain were visible by agarose gel electrophoresis (Kanhayuwa et al., 2015; Morley, 2017). However, in the present study we found a fifth dsRNA (dsRNA segment 5) and additional small bands were found by PAGE. Our sequence results indicated dsRNA 5 had a unique sequence, which has not previously been reported in the Af293 strain. We speculate AfuPmV-1 and AfuPmV-1M might share the same phylogenetic origin, but the fifth element has been lost in some branches of Af293 during passaging.

Since it was reported that some *A. fumigatus* strains, including the genome reference strain Af293, are infected with mycovirus, no studies have been performed to elucidate whether the mycovirus provokes hypovirulence of the host fungi in a mouse model (Bhatti et al., 2011; Kanhayuwa et al., 2015). For example, AfuPmV-1 had no statistically significant virulent effect (Kanhayuwa et al., 2015), while A78, a species related to AfuPmV-1, had a hypervirulence effect in an insect model (Özkan and Coutts, 2015).

Among the five AfuPmV-1M dsRNAs, the 5′ and 3′ untranslated regions (UTRs) have conserved nucleotide sequences, as is commonly found in RNA viruses with multipartite and multi-component genomes. This suggests that AfuPmV-1M does indeed possess five genomic dsRNA segments which replicate separately as a multipartite dsRNA mycovirus (Mertens and Sangar, 1985; Attoui et al., 1997). In the present study, the complete nucleotide sequences of the fifth dsRNA were newly determined as well as the sequences of the other four dsRNA sequences, which showed high similarity to the ones reported by Kanhayuwa et al. (2015). Each of the five dsRNAs was found to have a single ORF.

At present, the functions of the proteins encoded by the dsRNAs of segments 2–5 are unknown, except that the protein encoded by dsRNA 3 has the conserved motifs characteristic of a domain of S-adenosyl-L-methionine-dependent methyltransferases (AdoMet_MTases). AdoMet_MTases catalyze the transfer of methyl groups from the ubiquitous cofactor S-adenosyl-L-methionine to a multitude of biological targets with high specificity, including DNA, RNA, protein, polysaccharides, lipids, and a range of small molecules in fungal cells (Polevoda and Sherman, 2007). It is well-known that viral methyltransferases cause various diseases in host cells (Kharbanda et al., 2013); therefore, dsRNA 3 of AfuPmV-1M may play a role in reducing the virulence of its pathogenic host *A. fumigatus* in animal infections.

Through a series of experiments, we found additional dsRNA bands smaller than dsRNA 4. Sequence analysis revealed these are fragments of conjugated 5′- and 3′-sequences but they lack the middle parts of sequences in dsRNAs 1, 3, and 4. AfuPmV-1 has been reported to produce virus-derived small interfering RNA (vsiRNA) (Özkan et al., 2017). Inferring from this previous report, the smaller bands which occasionally appeared in the gel analysis of AfuPmV-1M may be remaining RNA fragments produced through such vsiRNA processing. Experiments are underway to elucidate the origin of these smaller sequences.

We have previously reported a double-stranded mycovirus, AfuCV41362, isolated from a different origin, of *A. fumigatus* strain IMF 41362 (Takahashi-Nakaguchi et al., 2020). AfuCV41362 consists of four dsRNA fragments and belongs to the *Chrysoviridae*. In that report we examined expression levels of viral RNA in host filamentous fungi at each developmental stage (to the best of our knowledge for the first time). The time course of AfuPmV-1M dsRNA expression was different from that of AfuCV41362 dsRNAs during host fungal development, as examined by real-time PCR (Takahashi-Nakaguchi et al., 2020). Similarly to AfuCV41362 dsRNAs, the expression levels of AfuPmV-1M dsRNAs increased as germination of the host fungus *A. fumigatus* proceeded. However, unlike AfuCV41362, expression levels of AfuPmV-1M viral RNAs were seen to increase at the time when the hyphae were completely extended. Also, the level of AfuPmV-1M RNAs increased at the time of spore maturation, whereas AfuCV41362 RNA increased only during the early stage of sporulation. This difference may be related to the fact that AfuCV41362 produces virus particles while AfuPmV-1M presumably does not (Kanhayuwa et al., 2015).

The present *in vivo* and *in vitro* experiments indicated that AfuPmV-1M virus-infected fungal strains have reduced pathogenicity in comparison with the pathogenicity of virus-free strains, based on the following results (summarized in Supplementary Table S3). (1) Mice infected with virus-infected fungi showed reduced mortality in comparison with the mortality of mice infected with virus-free fungi. (2) Lung tissues isolated from mice infected with the virus-infected strain showed fewer CFUs and less damage on a CT scan and histological images in comparison with the virus-free fungal strain. (3) Conidia of the dsRNA virus-infected strains showed reduced adherence to lung cells, reduced tolerance to macrophage phagocytosis, and reduced tolerance to oxidative and osmotic stresses. Furthermore (4), the growth of fungal hyphae and formation of conidia were retarded in the virus-infected strains in comparison with the virus-free strain during *in vitro* experiments. Incidentally, effects of AfuPmV-1M mycovirus infection on *A. fumigatus* were re-confirmed by introducing the mycovirus into the originally virus-free KU strain (AfS35, FGSC A1159, akuA:loxP) via the protoplast fusion method (Kanematsu et al., 2004; Lee et al., 2011; Takahashi-Nakaguchi et al., 2020). Tolerance to oxidative and osmotic stresses was decreased even in the whole virus-introduced strain (Supplementary Figure S8). On the other hand, in insect infection experiments AfuPmV-1 is reported to exhibit no pathogen-suppressing effect (Kanhayuwa et al., 2015). This may be due to differences between insects and mice or the action of the fifth ORF, ORF5.

The difference between the effects of AfuPmV-1M and AfuCV41362 was also observed in the RNA-seq results. With AfuCV41362 infection, mRNA expression in the host *A. fumigatus* was more severely affected at the time of germination (4 h swelling stage) than in the hyphal stage, but with AfuPmV-1M infection, mRNA expression of the host *A. fumigatus* was more severely affected at the hyphal stage, when viral RNA was highly expressed. Adhesion to host cells was reduced in virus-infected strain (Figure 6A). We previously revealed 23 genes expressed during sporulation that are important for adherence to host cells (Takahashi-Nakaguchi et al., 2017). Although the stages examined were different from the conidial maturation stage, the expression levels of three of the genes (*AFUA_1G13670*, conserved hypothetical protein; *AFUA_4G02805*, Asp hemolysin-like protein; and *AFUA_5G00590*, hypothetical protein) were reduced during germination, and the expression levels of four genes (*AFUA_4G09310*, conserved hypothetical protein; *AFUA_1G04100*, conserved hypothetical protein; *AFUA_4G01030*, conserved hypothetical protein; and *AFUA_8G07060*, hydrophobin, putative) were reduced at the mycelial stage in virus-infected strains. Host macrophages and neutrophils also produce high levels of reactive oxygen species (ROS), which are harmful to *A. fumigatus* (Brown et al., 2009). Multiple genes involved in the defense of *A. fumigatus* against ROS have been characterized, and the expression levels of several genes were reduced in the mycelium due to viral infection. These genes included *TcsC*, a sensor of oxidative stress (Chapeland-Leclerc et al., 2015), the stress response pathway gene *Pbs2*, and the gene encoding transcription factor AFUA_4G08120. In addition, the expression levels of osmotic stress sensor genes (*Msb1*, *Sho1*), which share oxidative stress and stress response pathways, were also down-regulated. Furthermore, the expression levels of 10 other stress response genes were also down-regulated by the virus in the mycelium (Supplementary Table S2). In particular, the expression of gliotoxin-related genes and gliotoxin production decreased and that of fumagillin-related genes and fumagillin production increased at the hyphal stage, a phenomenon not observed in the AfuCV41362 infected host (Takahashi-Nakaguchi et al., 2020). Gliotoxins have toxic and immunosuppressive properties against host immune effector cells, and has been implicated in virulence (Stanzani et al., 2005; Orciuolo et al., 2007; Sugui et al., 2007; Wang et al., 2014). It is presumed that the reduced production of gliotoxin caused by mycovirus is another factor that reduces the pathogenicity of the virus-infected fungus. In contrast to gliotoxin, gene expression levels and production of fumagillin, which is another toxin produced by *A. fumigatus*, were enhanced by the virus infection. Fumagillin is used as an antimicrobial agent and can block mammal blood vessel formation by binding to the enzyme methionine aminopeptidase 2; it is also toxic to erythrocytes at high doses (Guruceaga et al., 2019). At present, the effect of fumagillin in the pathogenicity of the fungal hosts remains unknown (Guruceaga et al., 2018).

To elucidate the role of each viral RNA, we expressed individual viral ORFs in the virus-free KU strain. The expression of ORF2 and ORF5 reduced the fungal burden in the lungs of host mice. In the whole AfuPmV-1M virus-infected strain a large amount of ORF2 was expressed during mycelial development and the sporulation stages, while colony growth and sporulation were severely defective in the ORF2-expressing strain. In addition, our data suggest that ORF2 suppresses fungal virulence by suppressing conidial adherence to lung cells and by reducing oxidative stress resistance. Although ORF5 suppressed osmotic tolerance, unlike ORF2, no other prominent virulence-suppressing effect was found in relation to ORF5. It should be noted that ORF5 is not present in AfuPmV-1, a closely related species that has previously been reported to have a low effect on suppressing fungal virulence in insects. The expression of ORF1, which contains RdRp, delayed the germination of host conidia. The expression of ORF3, which has a methyltransferase motif, resulted in abnormal colony morphology, delayed germination, enhanced phagocytosis by macrophages, and decreased oxidative stress and osmotic resistance, but did not affect the degree of fungal burden in mice. Although the expression level of ORF4 was high during the germination stages in whole-virus infected fungi, no significant effect on the fungal phenotypes was observed. Future analysis of each viral gene, under appropriate experimental conditions with controlled expression levels, should reveal the detailed roles of these viral components.

We conclude that the AfuPmV-1M mycovirus possess the ability to reduce fungal pathogenicity in a mouse infection model and suggest mycoviruses may provide novel information in the quest for novel therapeutic strategies for the treatment of aspergillosis. Further research aimed at the identification of mycovirus genes associated with reduced virulence of *A. fumigatus* is necessary to understand the underlying mechanisms and physiological roles of these genes. Such research may result in novel therapeutic biological agents for use against fungal infections.

## Data Availability Statement

The datasets generated for this study can be found in the online repositories. The names of the repository/repositories and accession number(s) can be found below: https://www.ncbi.nlm.nih.gov/genbank/, LC517041–LC517045; https://www.ncbi.nlm.nih.gov/genbank/, PRJDB9242.

## Ethics Statement

The animal study was reviewed and approved by the Institutional Animal Care and Use Committee of Chiba University.

## Author Contributions

AT-N and HM designed the experiments. AT-N, ES, MY, SU, KS, HC, AN, YC, and DH performed the experiments. AT-N, ES, and TG analyzed the data. AT-N and TG wrote the manuscript. All authors contributed to the article and approved the submitted version.

## Conflict of Interest

The authors declare that the research was conducted in the absence of any commercial or financial relationships that could be construed as a potential conflict of interest.

## Footnotes

1 https://talk.ictvonline.org/taxonomy/

2 https://elbe.hki-jena.de/fungifun/

3 https://www.genome.jp/tools/motif/
