# Supplementary material for: Phenotypic and Molecular Biological Analysis of Polymycovirus AfuPmV-1M From Aspergillus fumigatus: Reduced Fungal Virulence in a Mouse Infection Model
Source: Front Microbiol. 2020 Dec 11;11:607795. doi: 10.3389/fmicb.2020.607795 (PMC7794001; doi:10.3389/fmicb.2020.607795)
Supplement: Supplementary file 3 [file Data_Sheet_1.pdf]

Figure legends

Figure S1. (A) Multiple alignments of 5'- and 3'-untranslated regions (UTRs) of the five identified dsRNA segments with respect to the coding strand (ORF sense strand). Consensus sequences and conservation rates are shown below the nucleotide sequences. (B) RNA sequencing of the Af293 strain was performed, and mapped to the genome of AfPmV1-M. The reads were mapped except for 1 to 2 bases at the very tip, which are difficult to map, indicating that the genome sequencing was correct. We also confirmed the results by viral RNA sequencing using fragmented and primer ligated dsRNA sequencing (FLDS; Urayama et al., 2016; 2018).

Figure S2. Phylogenetic analysis of the putative RNA-dependent RNA polymerase (RdRp) sequences of AfuPmV-1M and other selected mycoviruses belonging to Chrysoviridae, Totiviridae, Partitiviridae, Reoviridae, and Polymycoviridae. The phylogenetic tree was generated using the neighbor-joining (NJ) method. Datasets were subjected to 1000 bootstrap replicates. The scale bars indicate the number of replacements per site. The numbers at the branch points indicate the bootstrap percentage. The full names and sequence information are given in Table S1.

Figure S3. (A) Comparison of dsRNA bands between the AfuPmV-1M-infected strain (lane V) and the virus-free strain (lane F) by agarose gel electrophoresis. Lane M, molecular weight marker. (B) Comparison of dsRNA amounts between the AfuPmV-1M-infected strain and the virus-free strain by RNA sequencing. For Virus-free strain all the expression were undetectable levels.

Figure S4. Correlation of differential gene expression between virus-infected (Virus) and virus-free (Free) strains analyzed by RNA-seq. A and B: Scatter plots of transcript levels (RPKMs) at the swelling stage (A: 4 h) and the hyphal stage (B: day 6). C: Transition of gene expression patterns in the virus-infected (circles) and virus-free (triangles) strains during the swelling stage (4 h, white) and hyphal stage (day 6, black). The pattern was examined using principal component analysis (PCA).

Figure S5. Functional catalog analysis of down-regulated (A) and up-regulated (B) genes at 4 h in the swelling stage and down-regulated (C) and up-regulated (D) genes on 6 day in conidia-forming stage. Common genes down-regulated (E) and up-regulated (F) at 4 h and day 6. The analysis was performed using the FungiFun web application (Priebe et al.,

2011). Genes which had more than two-fold reductions between the virus-infected and virus-free strains were analyzed. Detailed data underlying Figure S5 are attached as an MS Excel file (Table S2).

Figure S6. HPLC analysis of gliotoxin and fumagillin production by A. fumigatus on day 6 of the mycelial stage. Gliotoxin in the supernatant and fumagillin in mycelia were detected by absorbance at wavelengths of 254 and 330 nm, respectively.

Figure S7. Viral gene expression in each transformants during sporulation.
Figure S8. Comparisons of the whole-virus infected KU strain and the native virus-free KU. A-B. Radial growth of colonies. 2 strain of whole-virus infected KU strain (#2 and #3) were tested. C-D. Growth of each strain under stress conditions. Whole-virus infected KU strain #3 was used for these analysis.

Table S1. Information on the virus isolates used for sequence alignment and phylogenetic analysis of their RdRps.

Table S2. RNA-seq analysis data to compare gene expression between virus-infected and virus-free strains at two distinct time points: just before the start of swelling (4 h after the start of incubation, a stage referred to here as the 4-h swelling stage) and at the hyphal stage (day 6). (A) 4h down. Genes that are down-regulated in virus-infected strains compared to virus-free strains at 4h. (B) 4h Up. (C) 6days down. (D) 6days up. (E) Common genes down-regulated at 4h and day 6. (F) Common genes up-regulated at 4 h and day 6. Subsequently, GO data is also attached.

Table S3. Summary of results comparing phenotypic changes in A. fumigatus strains infected with the AfuPmV-1M virus (native virus) or transformed with plasmids by ectopic expression of each AfuPmV-1M ORF (ORF1–5). Downward and upward arrows indicate decreases and increases, respectively, in comparison with the respective control (virus-free strain or strain transformed with an empty vector). Effects are indicated only when statistically significant ($p < 0.05$).

Table S4. Primers used in the present study.

Figure S1A

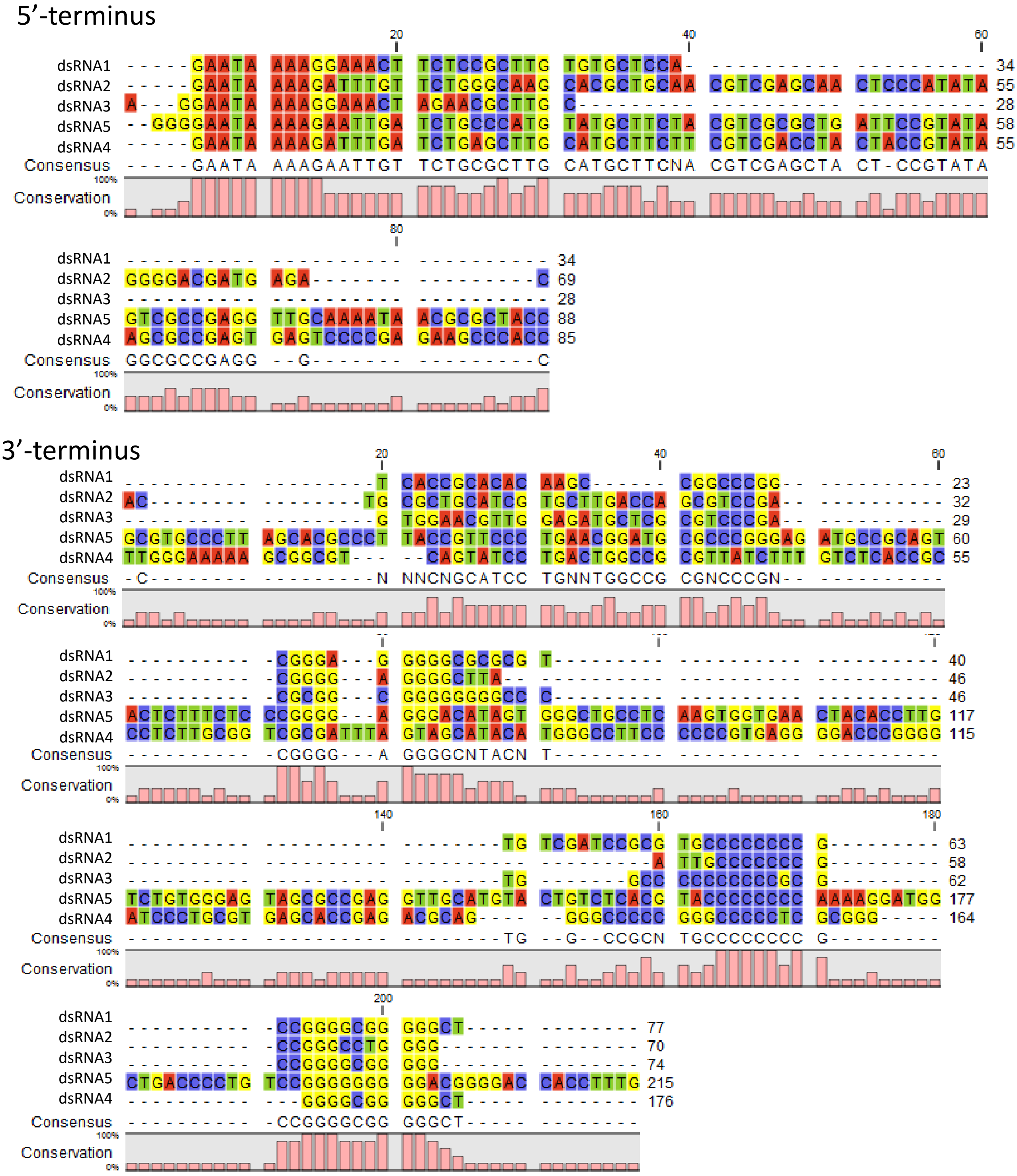

5'-terminus
dsRNA1
dsRNA2
dsRNA3
dsRNA5
dsRNA4
Consensus
Conservation
3'-terminus

Figure S1B

Name	Consensus length	Total read count	Average coverage	Reference length	Coverage length ratio (%)
AfuPmV1M1 mapping	2402	22865	999	2403	100.0
AfuPmV1M2 mapping	2230	67045	3058	2230	100.0
AfuPmV1M3 mapping	1970	33402	1755	1971	99.9
AfuPmV1M5 mapping	1156	9435	814	1156	100.0
AfuPmV1M4 mapping	1133	29937	2727	1140	99.4

Figure S2

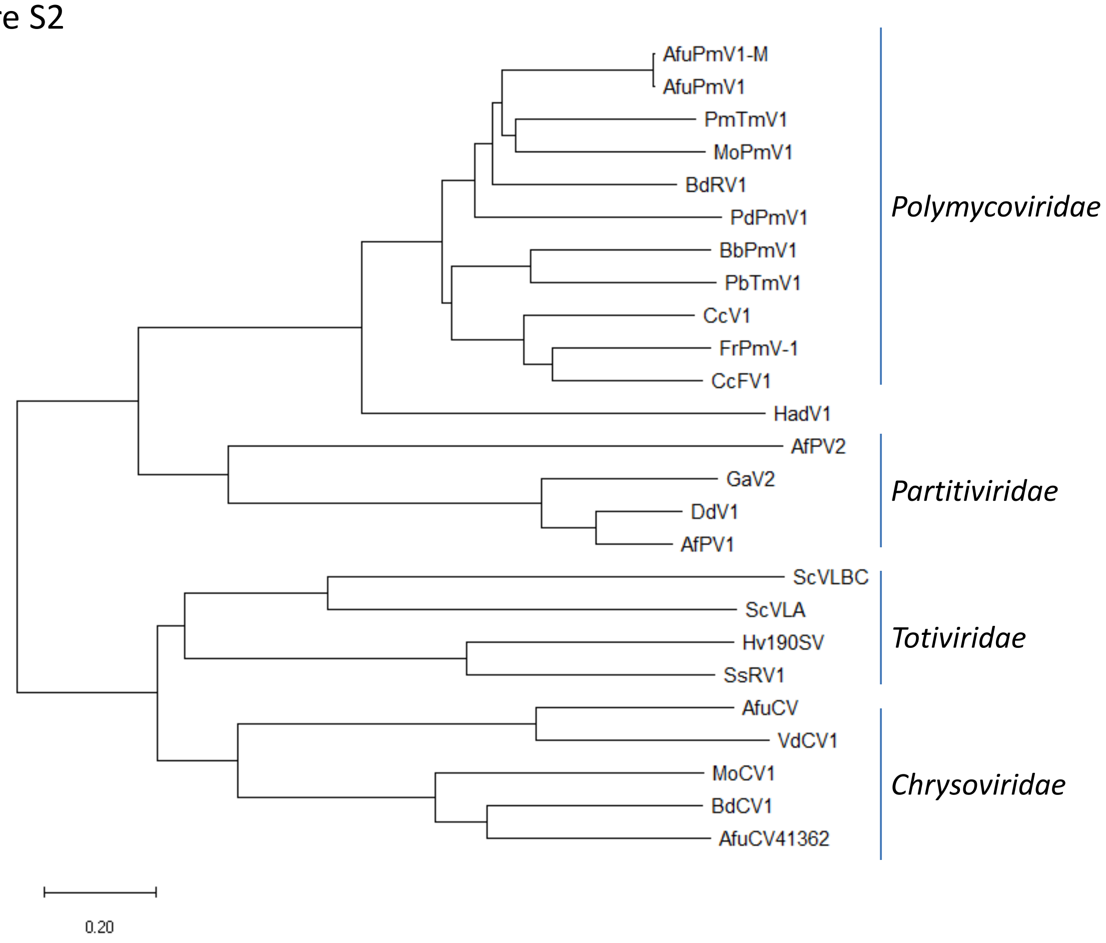

Table S1 Information on the virus isolates used for sequence alignment and phylogenetic analysis of their RdRps.

Virus name	Abbreviation	GenBank accession no.	Family
Aspergillus fumigatus chrysovirus	AfuCV	CAX48749	Chrysoviridae
AfuCV41362	AfuCV41362	LC350094	Chrysoviridae
Botryosphaeria dothidea chrysovirus 1	BdCV1	AGZ84312.1	Chrysoviridae
Magnaporthe oryzae chrysovirus 1	MoCV1	AB560761	Chrysoviridae
Verticillium dahliae chrysovirus 1	VdCV1	ADG21213.1	Chrysoviridae
Aspergillus fumigatus partitivirus 1	AfPV1	CAY25801	Partitiviridae
Aspergillus fumigatus partitivirus 2	AfPV2	YP_009553311	Partitiviridae
Discula destrucriva virus 1	DdV1	AAG59816	Partitiviridae
Grapevine associated Partitivirus-2	GaV2	ADU86896	Partitiviridae
Aspergillus spelaeus tetramycovirus 1	AsTmV1	AYP71805	Polymycovirus
Phaeoacremonium minimum tetramycovirus 1	PmTmV1	QDB74985	Polymycovirus
Aspergillus fumigatus polymycovirus-1	AfuPmV1	HG975302	Polymycovirus
Aspergillus fumigatus polymycovirus-1M	AfuPmV1-M	LC517041	Polymycovirus
Cladosporium cladosporioides virus 1	CcV1	YP_009052470	Polymycovirus
Penicillium brevicompactum tetramycovirus 1	PbTmV1	AYP71801	Polymycovirus
Colletotrichum camelliae filamentous virus 1	CcFV1	ASV63092	Polymycovirus
Beauveria bassiana polymycovirus 1	BbPmV1	CUS18595	Polymycovirus
Fusarium redolens polymycovirus 1	FrPmV1	QDH44656	Polymycovirus
Botryosphaeria dothidea virus 1	BdRV1	AKE49495	Polymycovirus
Magnaporthe oryzae polymycovirus 1	MoPmV1	QAU09249	Polymycovirus
Penicillium digitatum polymycoviruses 1	PdPmV1	YP_009551548	Polymycovirus
Helminthosporium victoriae virus 190S	Hv190SV	NP_619670	Totiviridae
Saccharomyces cerevisiae virus L-A	ScVLA	NP_620495	Totiviridae
Saccharomyces cerevisiae virus L-BC	ScVLBC	NP_042581	Totiviridae
Sphaeropsis sapinea RNA virus 1	SsRV1	NP_047558	Totiviridae
Hadaka virus 1	HadV1	BBU94038	Unclassified Riboviria

A

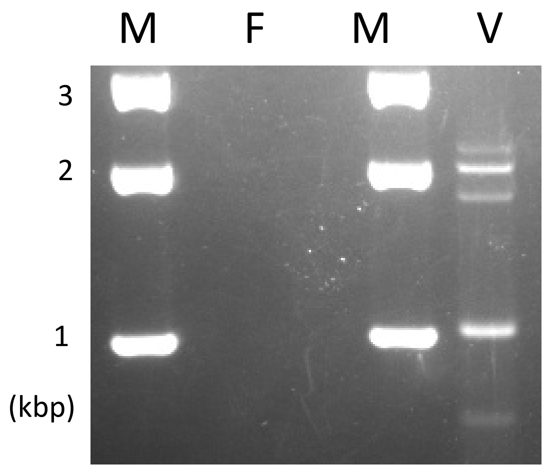

M
F
M
V
3
2
1
(kbp)

B

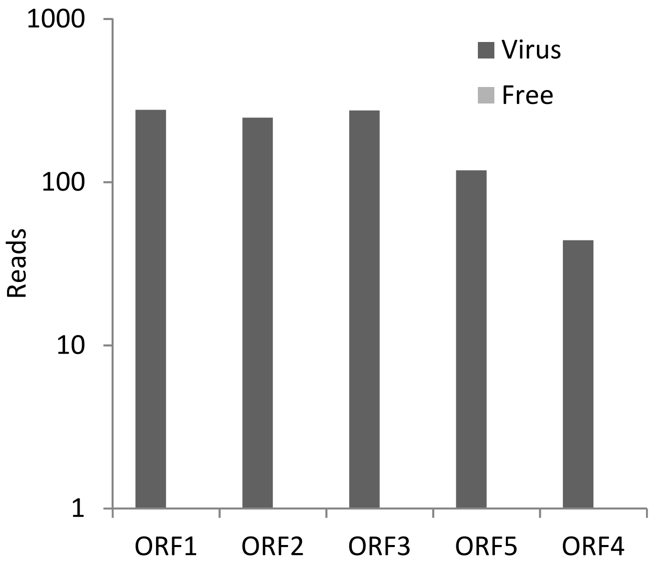

1000
100
10
1
Reads
Virus
Free
ORF1
ORF2
ORF3
ORF5
ORF4

Figure S3

A. Swelling stage (4 h)

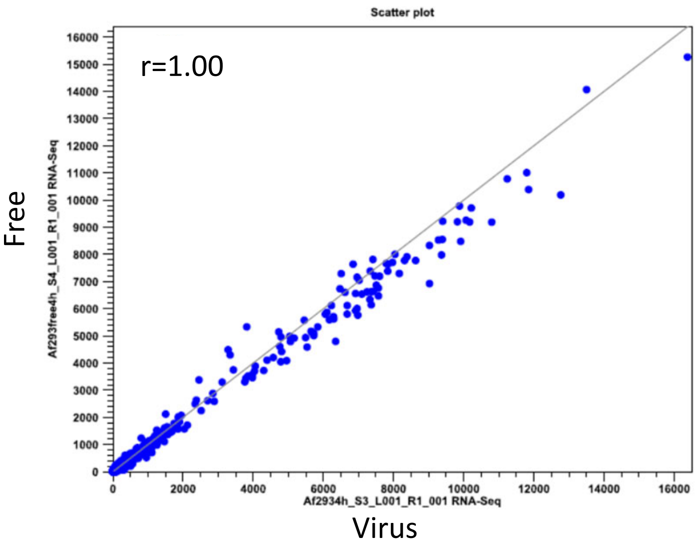

B. Hyphal stage (6 day)

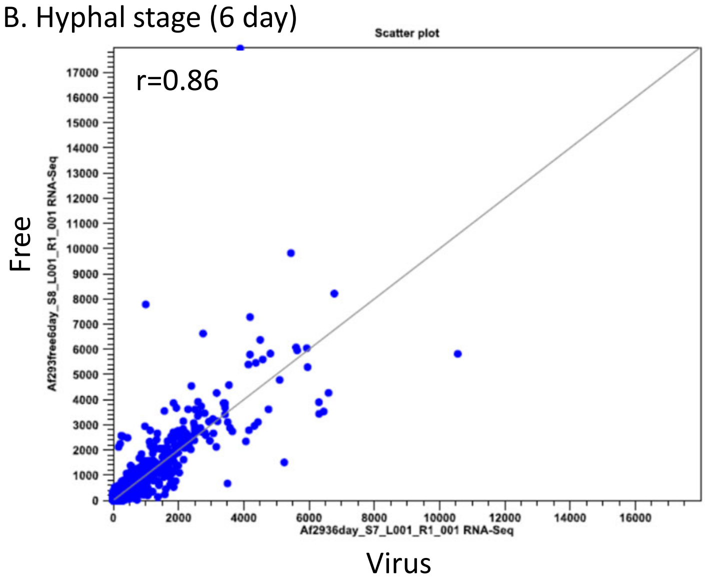

C. Principal component analysis (PCA)

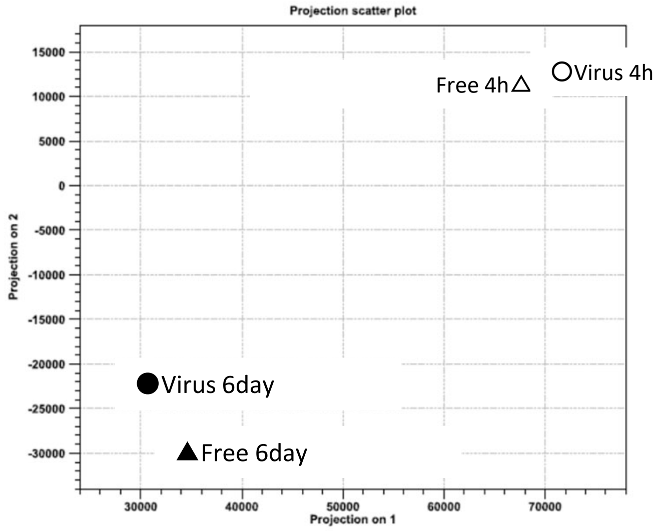

Figure S4.

Figure S5

A. 4h down

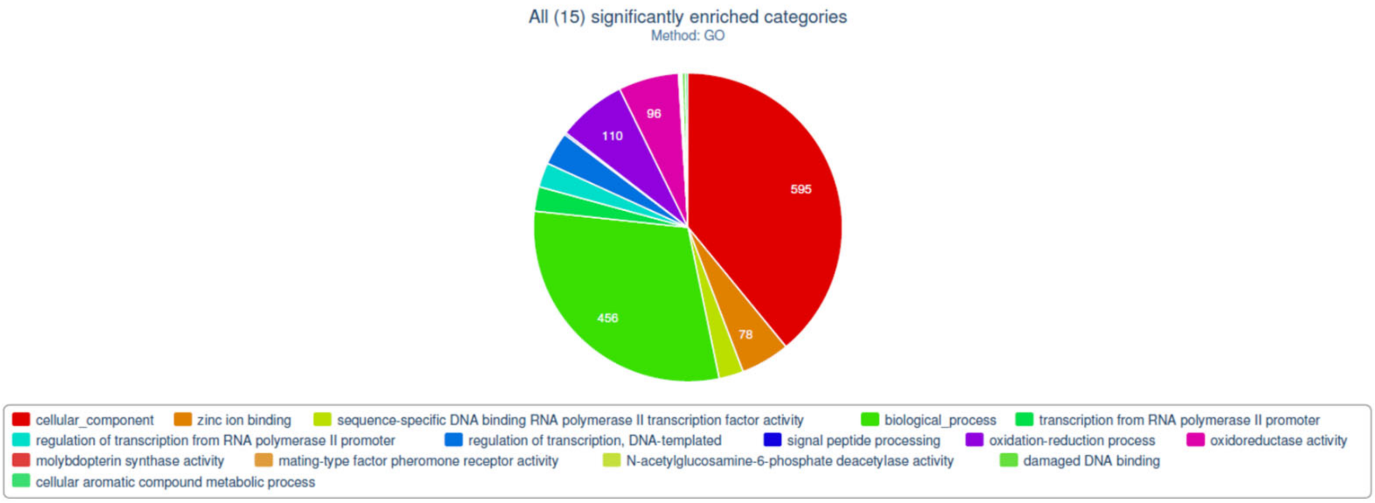

B. 4h up

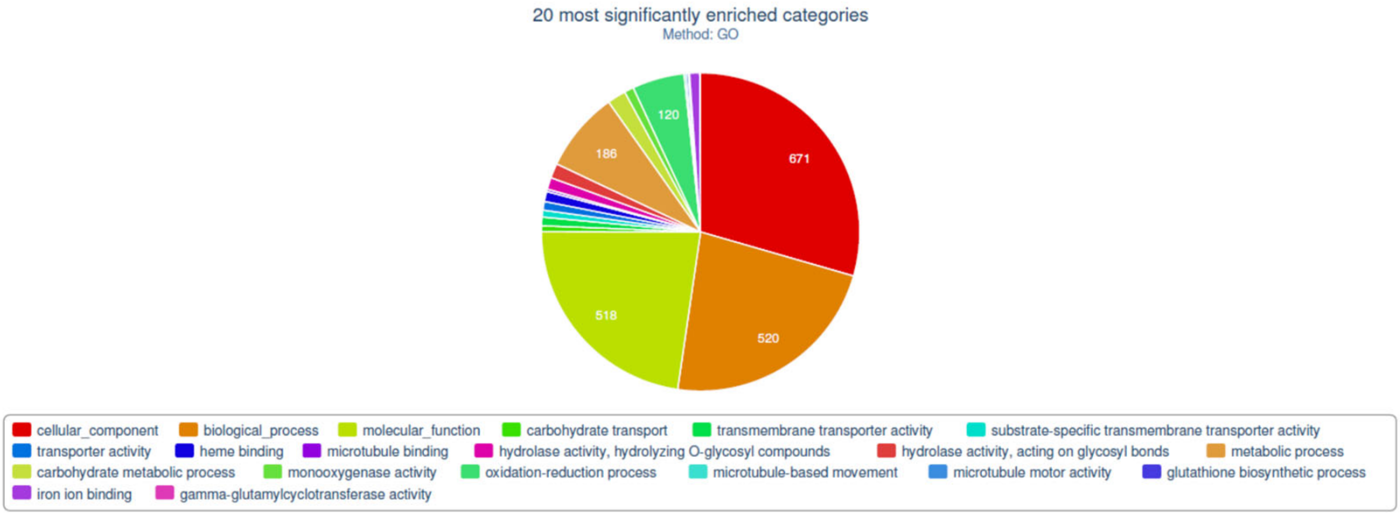

C. 6days down

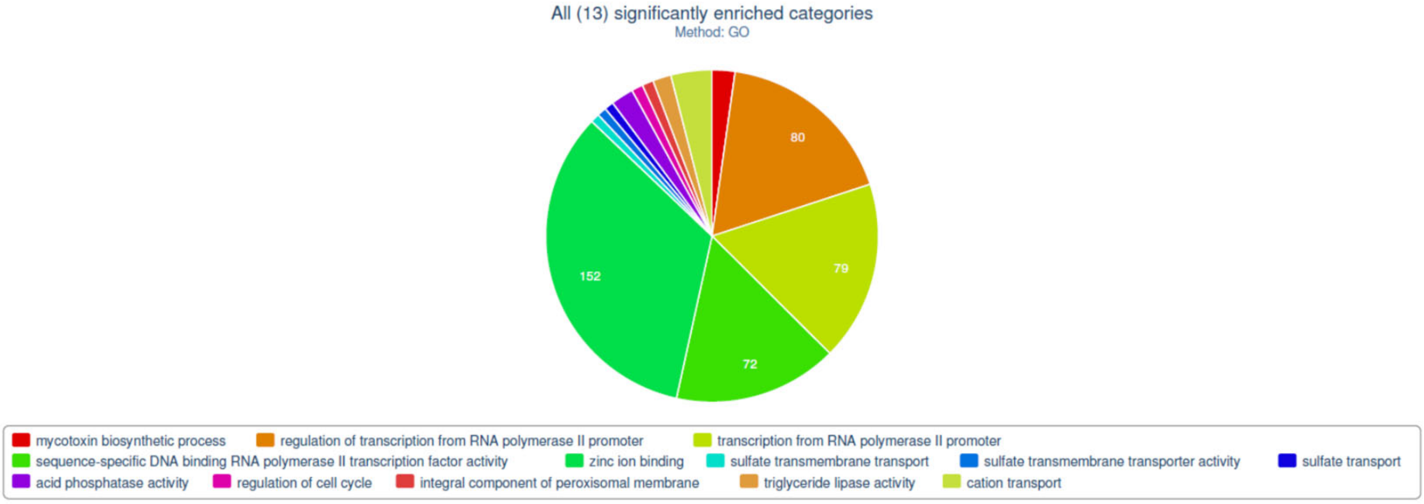

D. 6days up

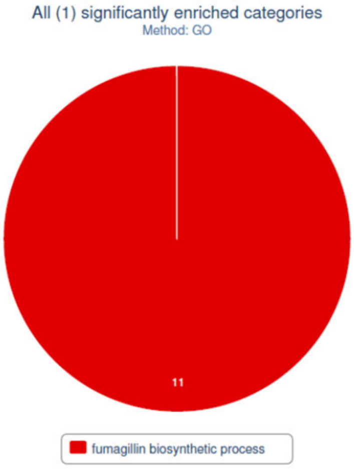

E. Common genes down-regulated at 4h and day 6.

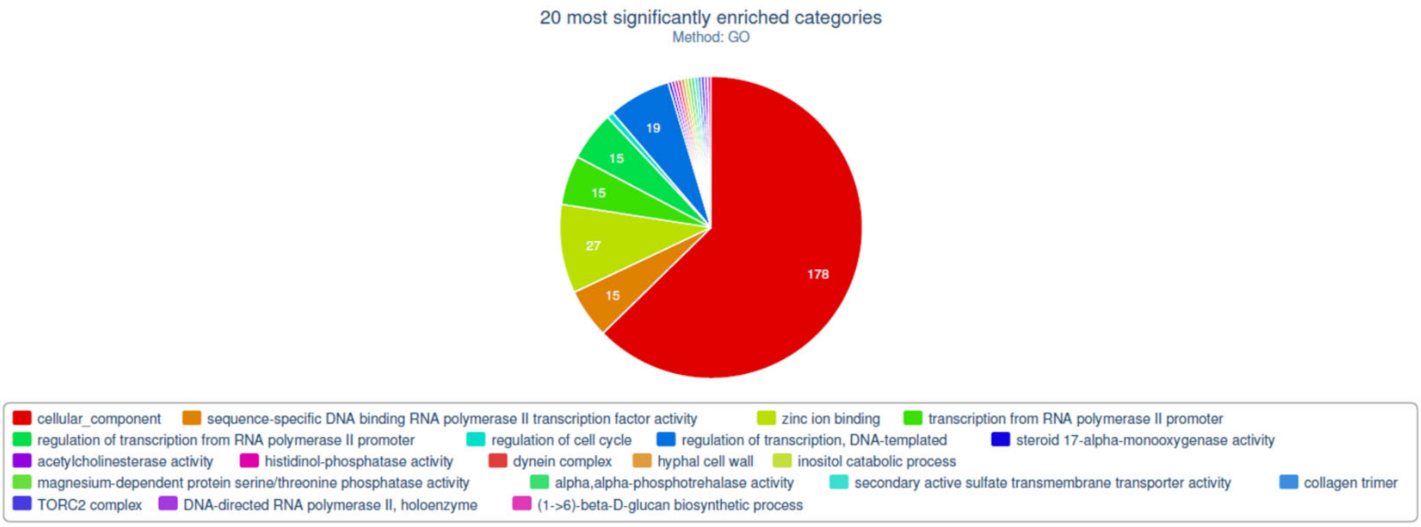

F. Common genes up-regulated at 4 h and day 6.

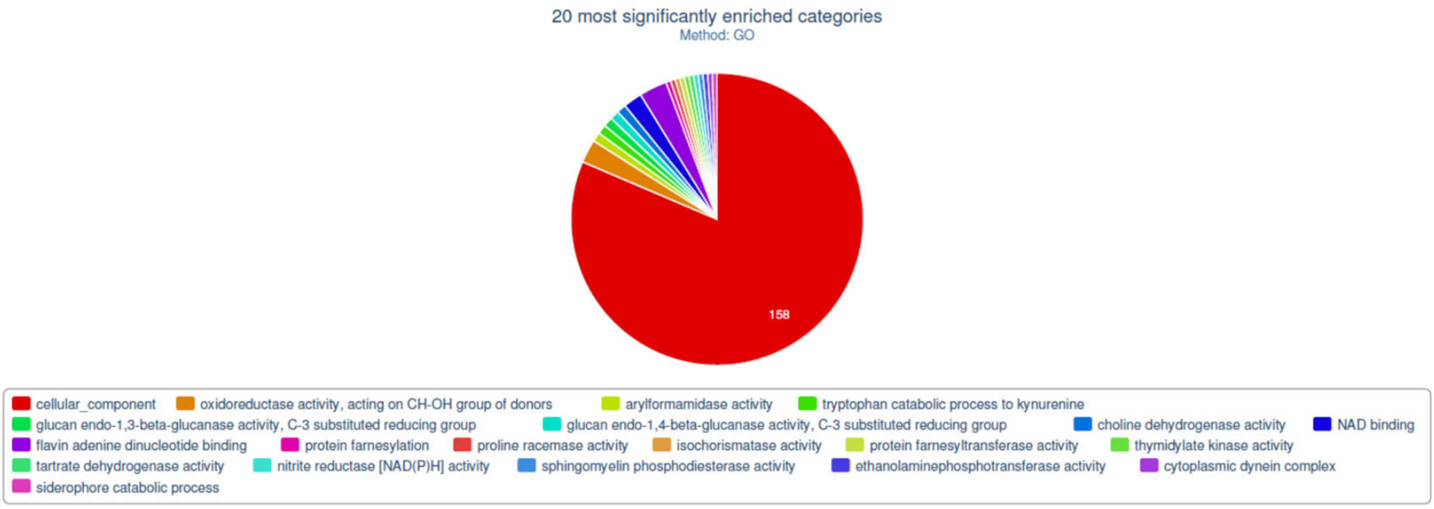

Figure S6

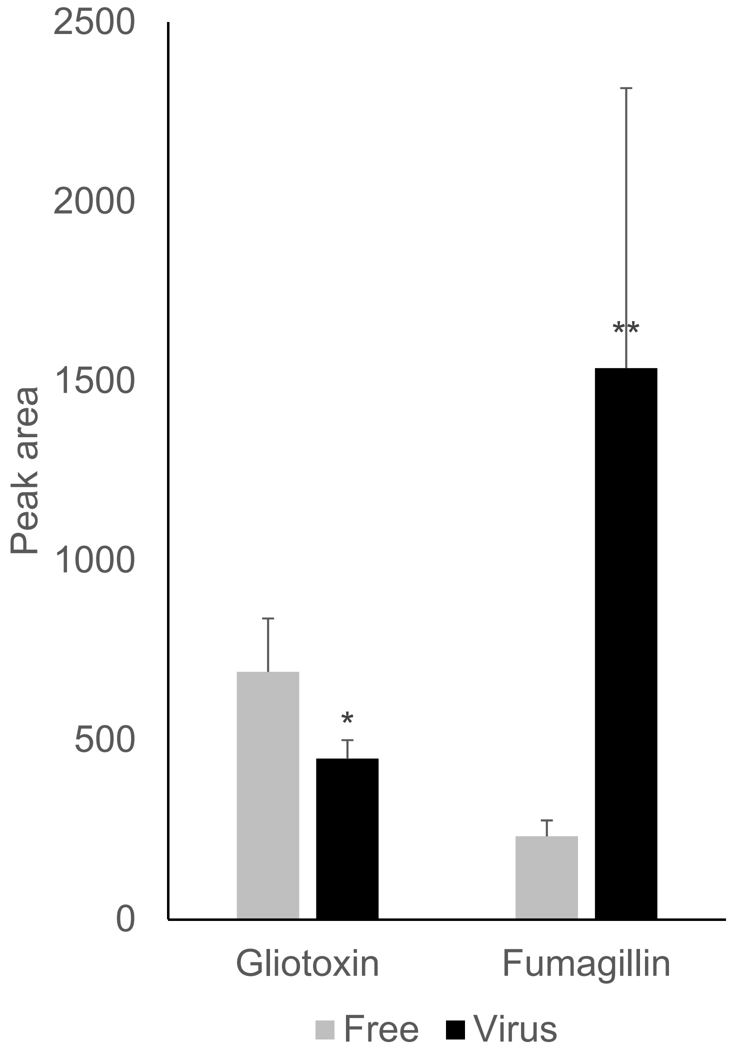
2500
2000
1500
1000
500
0
Peak area
**
*
Gliotoxin
Fumagillin
Free
Virus

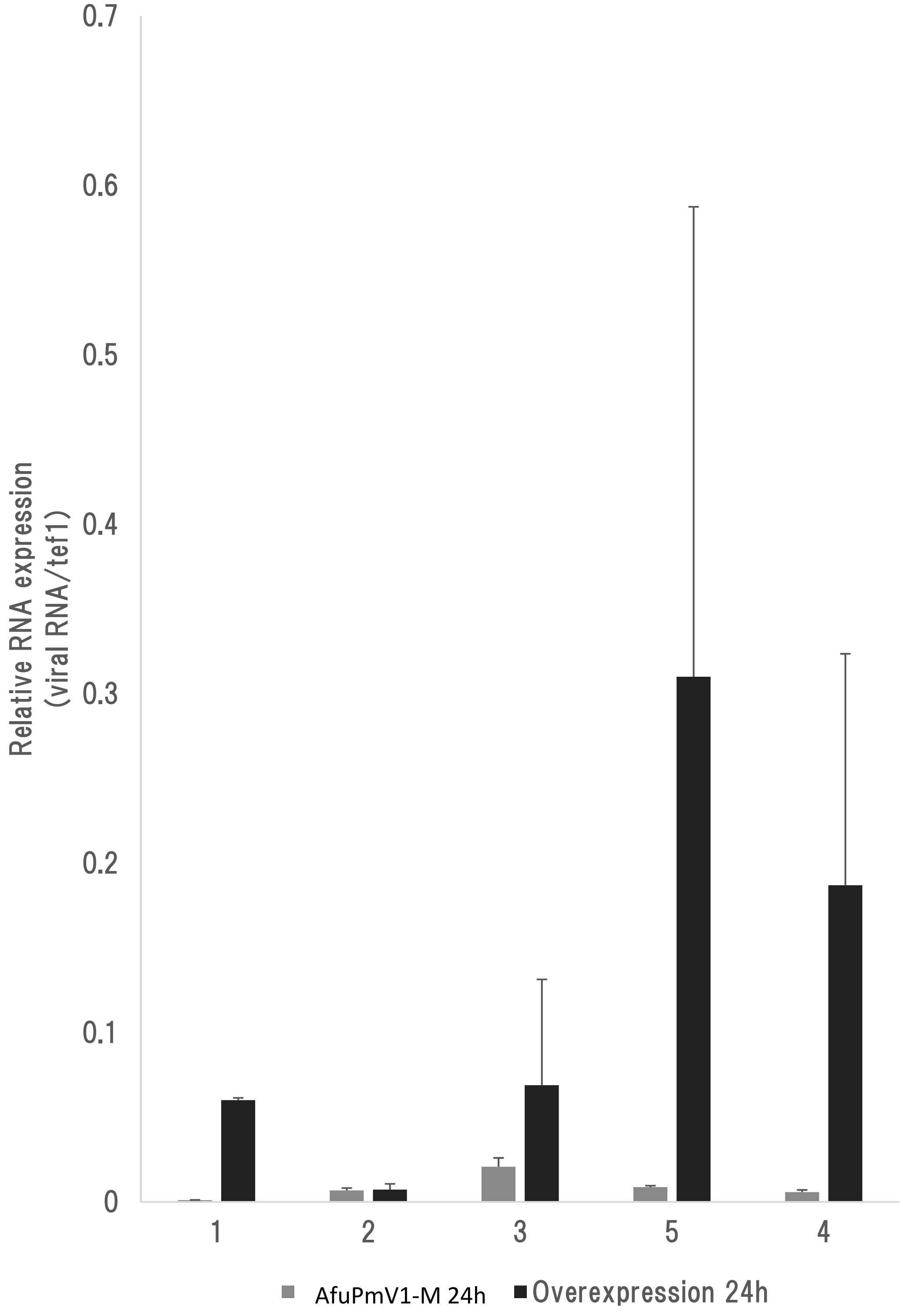
0.7
0.6
0.5
0.4
0.3
0.2
0.1
0
Relative RNA expression
(viral RNA/tef1)
1
2
3
5
4
AfuPmV1-M 24h
Overexpression 24h

Figure S7

Table S3

Parameters			Virus	ORF				
				1	2	3	5	4
Fungal phenotype	Influence at sporulation stage	Number of conidia	↓		↓			
		Tolerance to oxidative stress	↓		↓	↓		
		Tolerance to osmotic stress	↓			↓	↓	
		Adherence to lung cells	↓	↓	↓			
		Sensitivity to phagocytosis	↓			↓		
	Influence at germination stage	Swelling of conidia (6 h)	↓	↓		↓		
		Germination of conidia (8 h)	↓				↓	
	Influence to mycelial growth	Radial growth (on agar plate)	↓		↓	↓		
		Dry weight (in fluid medium)	↓					
		Damage to lung cells (LDH)				↑		↑
Influence to fungal virulence		Mouse lung CFU	↓		↓		↓	

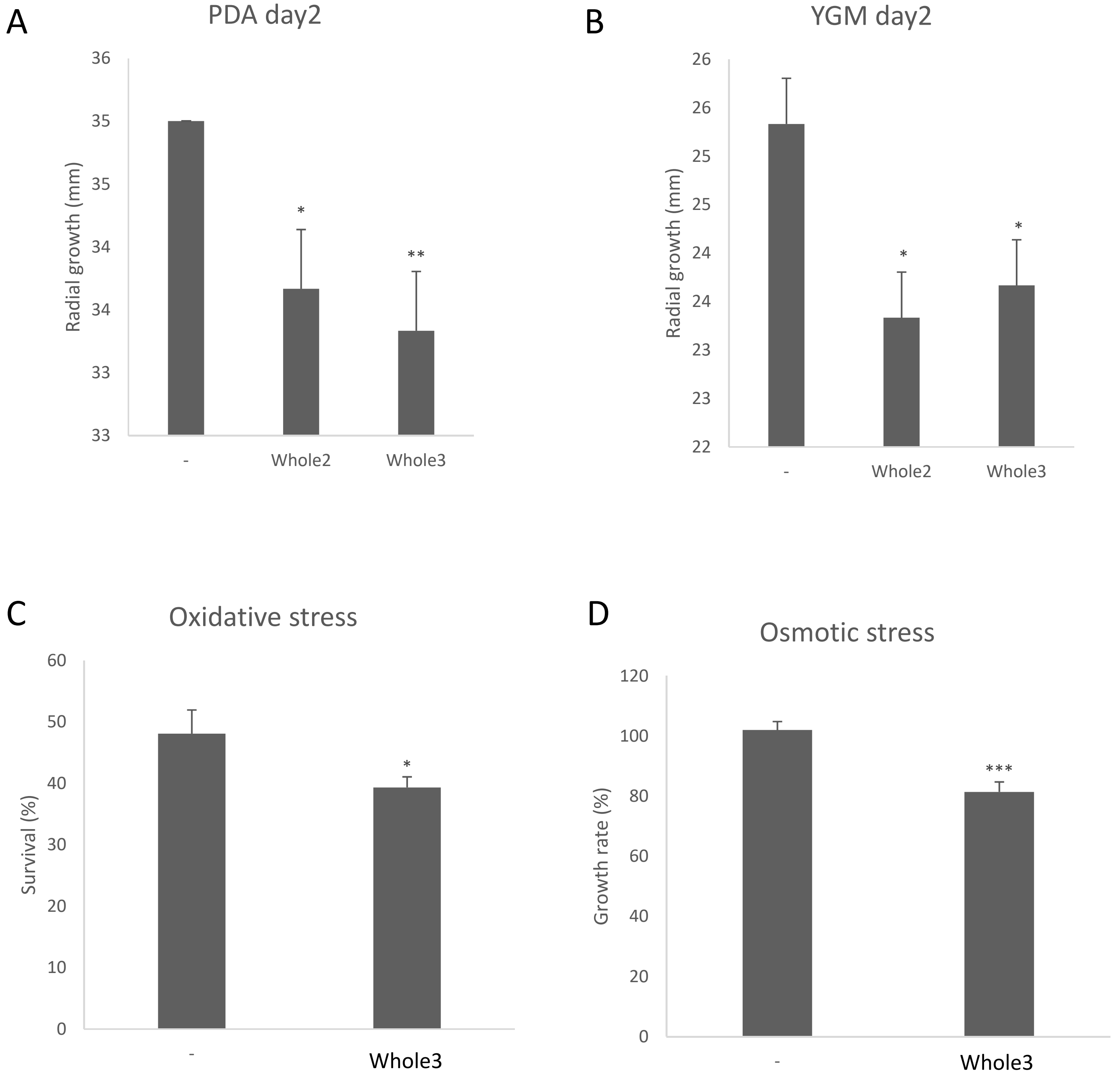
A
PDA day2
36
35
35
34
34
33
33
Radial growth (mm)
*
**
-
Whole2
Whole3
B
YGM day2
26
26
25
25
24
24
23
23
22
Radial growth (mm)
*
*
-
Whole2
Whole3
C
Oxidative stress
60
50
40
30
20
10
0
Survival (%)
*
-
Whole3
D
Osmotic stress
120
100
80
60
40
20
0
Growth rate (%)
***
-
Whole3

Figure S8
